# Prediction of Complications and Prognostication in Perioperative Medicine: A Systematic Review and PROBAST Assessment of Machine Learning Tools

**DOI:** 10.1097/ALN.0000000000004764

**Published:** 2024-01-01

**Authors:** Pietro Arina, Maciej R. Kaczorek, Daniel A. Hofmaenner, Walter Pisciotta, Patricia Refinetti, Mervyn Singer, Evangelos B. Mazomenos, John Whittle

**Affiliations:** Bloomsbury Institute of Intensive Care Medicine and Human Physiology and Performance Laboratory, Centre for Perioperative Medicine, Department of Targeted Intervention, University College London, London, United Kingdom; Wellcome/EPSRC Centre of Interventional and Surgical Sciences and Department of Medical Physics and Biomedical Engineering, University College London, London, United Kingdom; Bloomsbury Institute of Intensive Care Medicine, University College London, London, United Kingdom; and Institute of Intensive Care Medicine, University Hospital Zurich, Zurich, Switzerland; Bloomsbury Institute of Intensive Care Medicine, University College London, London, United Kingdom; Human Physiology and Performance Laboratory, Centre for Perioperative Medicine, Department of Targeted Intervention, University College London, London, United Kingdom; Bloomsbury Institute of Intensive Care Medicine, University College London, London, United Kingdom; Wellcome/EPSRC Centre of Interventional and Surgical Sciences and Department of Medical Physics and Biomedical Engineering, University College London, London, United Kingdom; Human Physiology and Performance Laboratory, Centre for Perioperative Medicine, Department of Targeted Intervention, University College London, London, United Kingdom

## Abstract

**Background:**

The utilization of artificial intelligence and machine learning as diagnostic and predictive tools in perioperative medicine holds great promise. Indeed, many studies have been performed in recent years to explore the potential. The purpose of this systematic review is to assess the current state of machine learning in perioperative medicine, its utility in prediction of complications and prognostication, and limitations related to bias and validation.

**Methods:**

A multidisciplinary team of clinicians and engineers conducted a systematic review using the Preferred Reporting Items for Systematic Review and Meta-Analysis (PRISMA) protocol. Multiple databases were searched, including Scopus, Cumulative Index to Nursing and Allied Health Literature (CINAHL), the Cochrane Library, PubMed, Medline, Embase, and Web of Science. The systematic review focused on study design, type of machine learning model used, validation techniques applied, and reported model performance on prediction of complications and prognostication. This review further classified outcomes and machine learning applications using an *ad hoc* classification system. The Prediction model Risk Of Bias Assessment Tool (PROBAST) was used to assess risk of bias and applicability of the studies.

**Results:**

A total of 103 studies were identified. The models reported in the literature were primarily based on single-center validations (75%), with only 13% being externally validated across multiple centers. Most of the mortality models demonstrated a limited ability to discriminate and classify effectively. The PROBAST assessment indicated a high risk of systematic errors in predicted outcomes and artificial intelligence or machine learning applications.

**Conclusions:**

The findings indicate that the development of this field is still in its early stages. This systematic review indicates that application of machine learning in perioperative medicine is still at an early stage. While many studies suggest potential utility, several key challenges must be first overcome before their introduction into clinical practice.

**P**erioperative medicine is a multidisciplinary specialty that focuses on meeting the complex medical needs of patients at risk of complications from surgery. With the number of surgical operations worldwide expected to rise to 500 million by the end of the 21st century,^1,2^ there is a growing need to accurately identify patients at risk and to manage potential complications. The incidence of postoperative mortality ranges from 1.7 to 5.7%^3–6^ and accounts for 7.7% of the global burden of death.^9^ Postoperative morbidity represents a major issue, with 16% of patients developing serious complications.^5,7,8^ This can affect both quality and length of life, placing a significant burden on individuals, families, and the healthcare system.^9–14^

Over the last 10 yr, there has been an emergence of novel predictive tools for perioperative outcomes driven by artificial intelligence and machine learning techniques. These tools offer exciting opportunities for advancing perioperative medicine. However, effective implementation requires a comprehensive understanding of both their advantages and potential risks. Machine learning, a subset of artificial intelligence, relies on algorithms to make predictions or decisions without explicit programming. Machine learning can analyze large, intricate data sets, learn from the data, and improve its performance over time. By enabling more accurate risk prediction as well as personalized treatment plans, machine learning has the potential to enhance patient care and outcomes. Nonetheless, well founded concerns currently exist regarding bias, interpretability, and reproducibility.

The distinction between classical statistics and machine learning can be blurred as they share common techniques including the development of risk scores.^15^ Existing risk stratification tools such as POSSUM, SORT, and NELA have traditionally utilized logistic regression, a statistical technique also employed in machine learning for similar purposes.^16^ However, classical statistics may struggle with nonlinear relationships and large numbers of variables, whereas the advantage of machine learning lies in its diverse range of algorithms that can model complex relationships and perform variable selection.^17^

We herein report a systematic review that focuses on prognostic artificial intelligence and machine learning models in perioperative medicine, aiming to carefully appraise the literature and identify knowledge gaps. Bias was evaluated using the Prediction model Risk Of Bias Assessment Tool (PROBAST),^18^ and an *ad hoc* classification was developed to determine the readiness level of the machine learning algorithms reported. We narrowed the scope of this systematic review to include only those studies that explicitly utilized machine learning approaches. Risk stratification tools based solely on logistic regression, commonly used as clinical benchmarks, were not included. For an analysis of these scores, readers are directed to a separate review.^16^

## Materials and Methods

This systematic review was structured according to the 2020 Preferred Reporting Items for Systematic Review and Meta-Analysis (PRISMA) protocols statement.^19^ The protocol was registered with the International Prospective Register of Systematic Reviews (CRD42022345213).

**Fig. 1:**
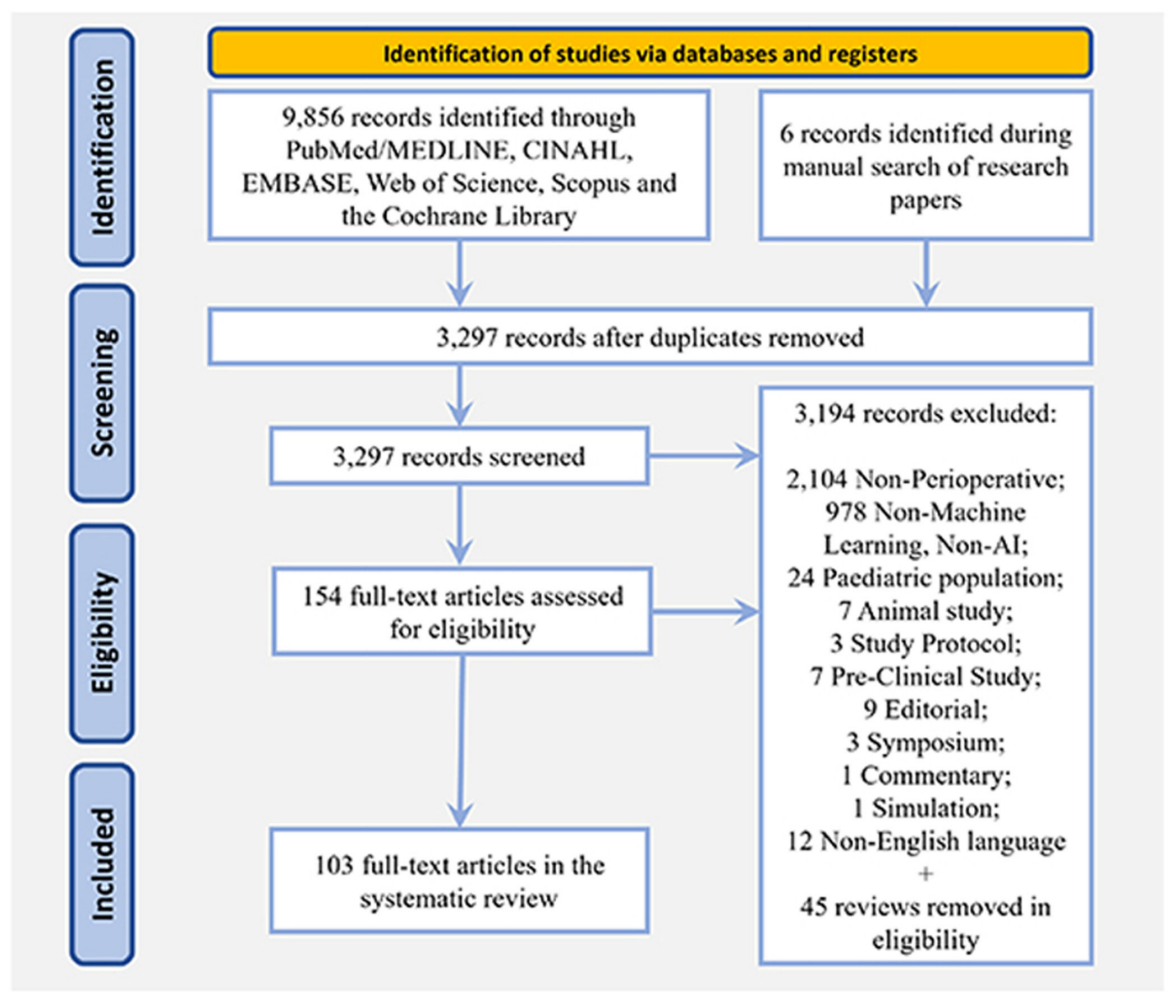
Preferred Reporting Items for Systematic Review and Meta-Analysis (PRISMA) 2020 flow diagram for new systematic reviews, including searches of databases and registries.

A literature search was conducted using Scopus, Cumulative Index to Nursing and Allied Health Literature (CINAHL), the Cochrane Library, PubMed, Medline, Embase, and Web of Science and completed on August 8, 2023. A primary search strategy was developed creating strings of research including the following keywords: “artificial intelligence,” “machine learning,” “preoperative,” “perioperative,”“surgery,”“anesthesia.”The detailed research query is described in the appendix. Search results were imported into EndNote 20 (Clarivate, United Kingdom). To assess the eligibility of the studies we used the Transparent Reporting of a multivariable prediction model for Individual Prognosis Of Diagnosis (TRIPOD) checklist (fig. 1).^20^

A multidisciplinary team of six reviewers assessed articles for eligibility, screening titles and abstracts to ensure relevance and identifying articles for full-text review. Each study was assessed by the reviewers independently. Two independent groups composed of two reviewers each (P.A., M.R.K. and D.A.H., W.P.) screened the full text to ensure each article was eligible following our inclusion and exclusion criteria. Conflicts were resolved by reviewer consensus.

We included retrospective and prospective studies in adult patients (18 yr old or older) published in the English language between January 1, 2000, and August 8, 2023. Outcomes of interest comprised but were not limited to:

### 1 Mortality

- Perioperative mortality risk

### 2 Morbidity

- Anesthesia risk
  - Risk of difficult/failed intubation
  - Need for massive transfusion
- Intraoperative complications
  - Bradycardia
  - Hypotension
  - Other potential complications
- Postoperative complications
  - Sepsis
  - Respiratory failure
  - Cardiovascular failure
  - Renal failure
  - Ileus
  - Soft tissue, skin, or wound infections
  - Delirium
  - Pain

### 3 Process

- Need for intensive care unit admission
- Length of hospital stay
- Overnight hospital stay
- Readmission to hospital

Exclusion criteria included pediatric populations, non-English language articles, protocol studies, symposium papers, studies conducted on animal models, *in vitro* studies, non–perioperative-focused studies, and studies unrelated to machine learning or artificial intelligence.

Study quality was assessed using established methodologies. To assess study quality and the readiness level of the machine learning algorithms, the authors created agreed *ad hoc* criteria (table 1; supplementary table 1, https://links.lww.com/ALN/D308).This is the recommended approach to assessing heterogenous nonrandomized clinical trials.^21^ The grading describes the readiness level of each machine learning model for possible clinical application, the type of study conducted, and the degree of validation.

**Fig. 2:**
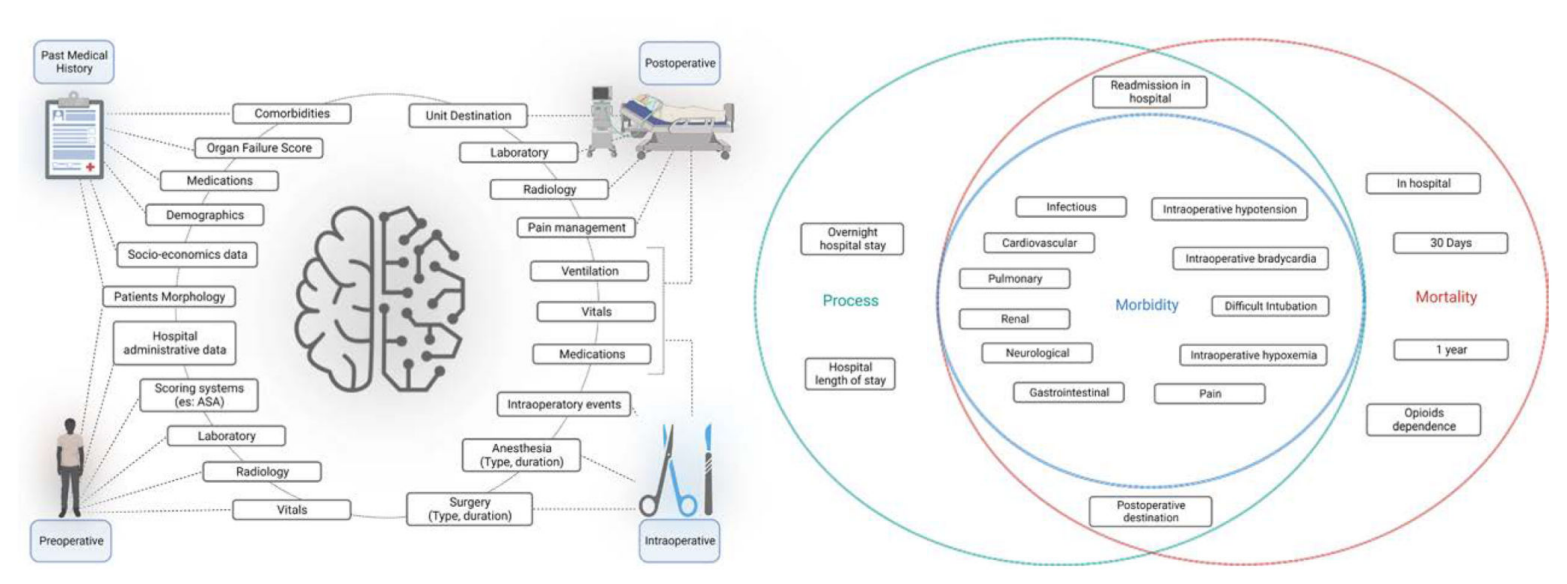
(*Left*) Graphical description representing the four feature categories used in perioperative medicine. (*Right*) Venn diagram of outcomes predicted in perioperative medicine. *Green*, process-related outcomes; *blue*, morbidity-related outcomes; *red*, mortality-related outcomes. ASA, American Society of Anesthesiologists.

**Fig. 3:**
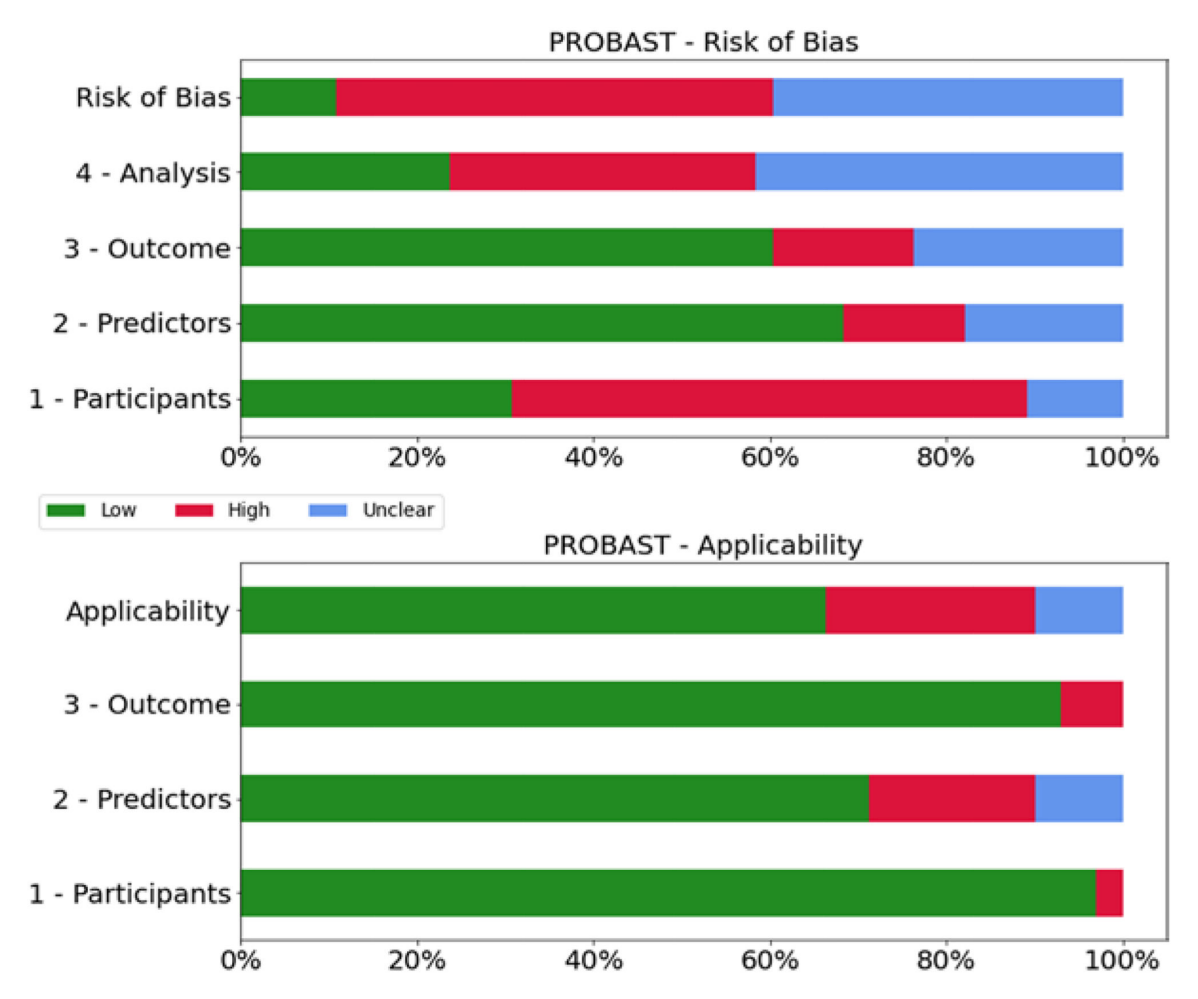
Prediction model Risk Of Bias ASsessment Tool (PROBAST) assessment.

Five authors (P.A., M.R.K., D.A.H., W.P., and P.R.) independently assessed the quality of studies that met the inclusion criteria using the PROBAST to review all prognostic artificial intelligence and machine learning models developed or validated in perioperative medicine (fig. 2 and fig. 3). Cohen’s κ agreement between authors was calculated.^22^ This tool evaluates the risk of bias in studies across four domains: participants, predictors, outcome, and analytic technique. The applicability of each study to the search question was assessed by evaluating its relevance to the specified population, predictors and outcomes.^18,23^ A score was assigned to each study based on this tool.^24^ A PROBAST is being specifically developed to assess artificial intelligence and machine learning models (PROBAST–artificial intelligence)^25^ but was not available at the time of study.

The data were extracted into tables by the two groups of two reviewers and cross-referenced to identify possible errors. Variables were extracted and tabulated in Excel (Office 365, Microsoft, USA), summarizing study content and using standard terminology (supplementary table 1, https://links.lww.com/ALN/D308). The best area under the receiver operating characteristic curve (AUC) and metrics available for each described model were recorded. The AUC values were included as part of our study reporting and analysis rather than for the purpose of comparing performance between different studies or models. The values were expressed as mean or median, as appropriate. Summary data were used to produce the figures and tables describing the different studies.

## Results

### Study Selection

An initial search identified 9,856 articles that satisfied our criteria (fig. 1). After removal of duplicates, 3,297 articles were retained; of these, 154 full-text articles were assessed for eligibility, of which 103 studies met the inclusion criteria. These studies are summarized in table 2 and supplementary table 1 (https://links.lww.com/ALN/D308), including study design, patient populations, outcomes (or target variables), machine learning models applied, model performance and validations, and study limitations. Most of the studies were published in 2021 or 2022. Studies predominantly originated from the United States (48), the People’s Republic of China (22), and South Korea (12).

### Study Types

A total of 63 studies were retrospective single-center: 13 were retrospective multicenter, and 11 employed retrospective analyses of national databases. Only 10 studies were prospective single-center, and 2 were prospective multicenter.^40^ One study utilized both a retrospective database and enrolled patients for a prospective study,^89^ while another used a cross-sectional study design.^58^ Two studies were secondary analyses of previous research, and one article described a specific model.^41^ There were 85 internal databases used to develop and evaluate the different models. External validation was performed in 12 studies.^42,74,78,125^ As reported in supplementary table 1 (https://links.lww.com/ALN/D308), most studies utilized tabular data, whereas those that predicted real-time events employed time-series analysis. A single study employed an image database.^72^

### Risk of Bias Assessment with the PROBAST

The results are summarized in supplementary table 2 (https://links.lww.com/ALN/D309) and figure 3. Cohen’s κ agreement among authors averaged 0.71, indicating substantial agreement. Most studies described the development of prognostic models. Of all the articles, 90% were rated as having a high or uncertain risk of bias. The predominant reasons for the high or unclear risk of bias in the analysis domain were the lack of timely and accurate description of model metrics, insufficient or unclear number of events per predictor included in the model, and/or unclear assessment of overfitting correction and adaptation. In the participant domain, patient selection was often not clearly stated, nor was there a clear description of inclusion and exclusion criteria, leading to a high risk of bias in 75% of studies. The overall risk of bias was high, as almost 90% of the articles did not present external validation. With respect to applicability, the predictor domain had the highest level of possible bias. Several studies used particular features such as insurance codes to identify procedures or utilized medical intraoperative data such as continuous electroencephalography (EEG) monitoring that are not routinely collected and may not be broadly available.

### Machine Learning Model Development Stage

**Fig. 4:**
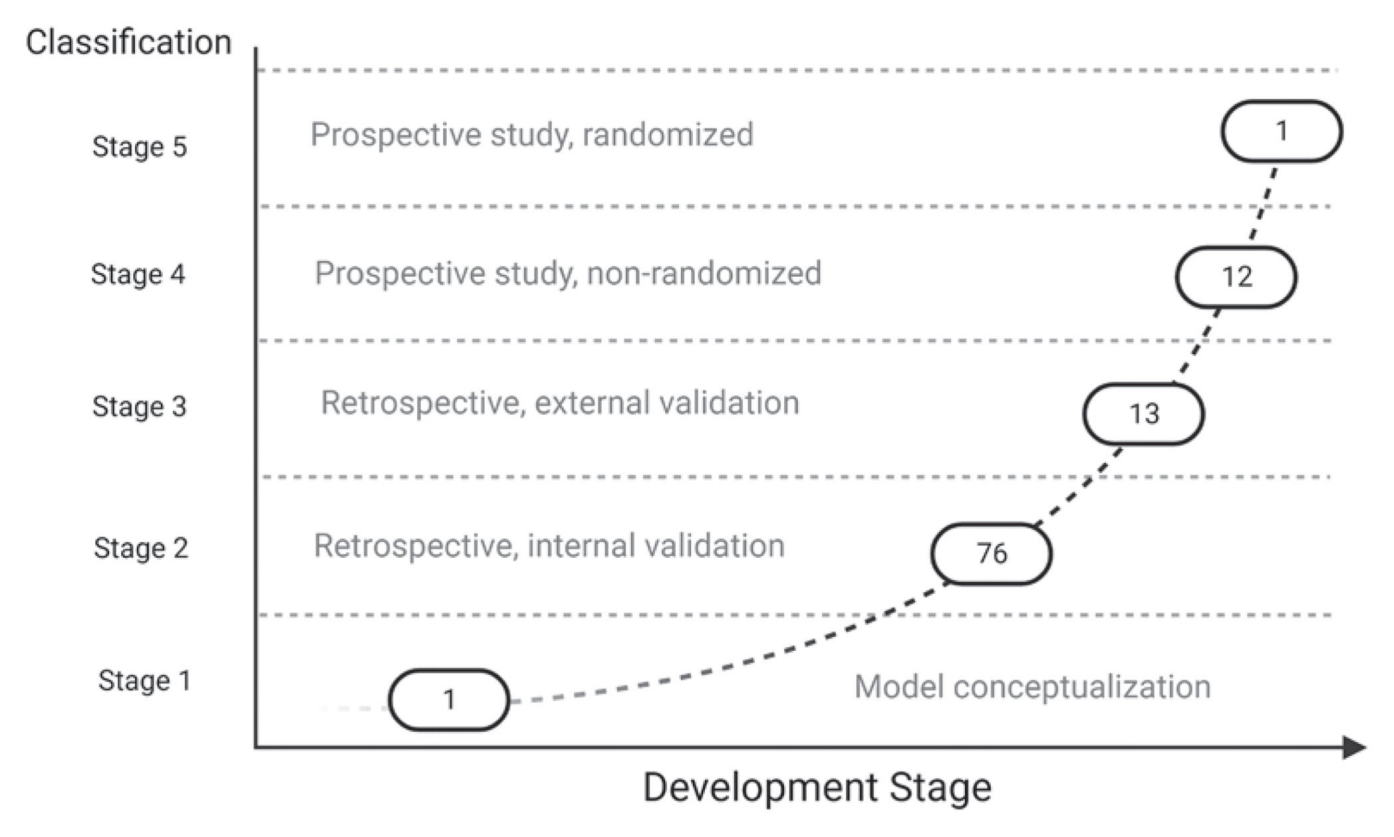
Graphical representation of the number of articles divided by the clinical development stage according to our *ad hoc* classification method.

Using our *ad hoc* classification (table 1 and fig. 4) to assess study quality and to quantify the development and implementation of machine learning models in perioperative medicine (supplementary table 2, https://links.lww.com/ALN/D309; figure 4), one study (1%) was classified as stage 1 or pre−model conceptualization;^41^ 76 (74%) were classified as stage 2 or model developed using a retrospective data set with internal validation; 13 (13%) were classified as stage 3, or models developed using retrospective study but with external validation;^58,78,125^ and 12 (11%) as stage 4, a model trained over prospective studies with internal validation. Only one (1%) study achieved a stage 5 grading. This was a prospective study with randomized control trial characteristics; however, it was conducted unblinded and limited to only 68 patients.^90^

### Model Validation Methods

All studies performed internal validation (supplementary table 1, https://links.lww.com/ALN/D308), albeit using different approaches. A total of 10 studies did not state the method of validation, while 36 performed multiple-fold cross-validation. The remainder used a hold-out method, typically using a training:test ratio of 70%:30% or 80%:20%. External validation utilizes databases that are completely independent from the one used to create the model. However, only 14 (13%) studies applied external validation,^26,27,42,52,58,74,78,80,83,98,112,114,126^ while the remaining 87% used only internal validation.

### Machine Learning Algorithms

Most studies reported their model performance using standardized classification metrics, namely sensitivity, specificity, accuracy, Brier score, area under precision recall, and F1 score.^127^ All models reported AUC (or C-statistic), a measure of the ability of a classifier to distinguish between two classes.^128^ For regression, the metrics reported were mean squared error and mean absolute error. Supplementary table 1 (https://links.lww.com/ALN/D308) shows all evaluation metrics of the models and the best performing model reported for each study.

### Outcomes

There was a high level of heterogeneity in the application of artificial intelligence and machine learning to perioperative medicine, as shown by the wide range of outcomes studied.The main outcomes are categorized by type of surgery (table 2). The type of outcome and features used are shown in figure 2, and the details of the machine learning models are reported in supplementary table 1 (https://links.lww.com/ALN/D308).

### Morbidity

Morbidity outcomes include deviations from normal patient trajectories in the postoperative period, *e.g*., development of kidney failure or delirium. Given the substantial volume of studies in this area, the outcomes were further categorized based on the typology of the potential clinical tool and the type of data used:

- Prognostication models: designed to predict the risk of adverse events, complications, or other negative outcomes in patients, based on tabular data.
- Real-time prediction models: designed to aid clinicians in making decisions during surgery or other medical interventions during clinical operations, usually based on time-series data.

There were 89 studies describing different types of morbidities. Fuller details of model performances are shown in supplementary table 1 (https://links.lww.com/ALN/D308).

#### Prognostication Models

These studies created models to stratify patients into different risk levels during the perioperative period using tabular data exported by electronic health record systems or obtained from national databases such as the American College of Surgeons−National Surgical Quality Improvement Program database.^129^ The main features used to run these models were demographic and socioeconomic data, diagnosis, medical history, scores such as American Society of Anesthesiologists (ASA) status or Charlson Comorbidity index, type of anesthesia, type of surgery, duration of surgery, training level of the surgeon, and clamp time.^130^

- Anesthetic and surgical risks: Several models were developed to predict anesthetic risk,^64^ risk of postinduction hypotension,^124^ identifying patients at risk of obstructive sleep apnea,^79^ or risk of postoperative re-intubation.^43,84^ Tavolara *et al*.^72^ used an online database comprising thousands of celebrity faces to train a neural network model to predict the risk of difficult intubation using a standardized picture of the patient.
- Development of postoperative complications: Most studies focused on the prediction of postoperative acute complications^26–28,42,44–49,59,99,110,112,113,118,123^ such as pain and opioid use,^53–57^ postoperative atrial fibrillation (new-onset atrial fibrillation),^82^ postoperative risk of stroke or myocardial infarction,^50,71,77^ and delirium or cognitive decline.^65–70^ Other models focused on the risk of developing pneumonia or respiratory failure,^83,85,125^ acute kidney injury,^43,52,58,60–63,120–122^ liver failure^117^ or development of sepsis or surgical site infection.^74–76,87,99,131^ Suhre *et al*.^78^ analyzed the association between perioperative nausea and vomiting and cannabis use using a long-term survey.
- Transfusion need and blood pressure prediction: Three studies developed models to predict the risk of perioperative transfusion in general surgery^80,81^ and cardiac surgery.^119^ Tan *et al*.^51^ developed a model to predict early phase postoperative hypertension after carotid endarterectomy. Hatib *et al*.^89^ used preprocessed data from continuous arterial monitoring obtained during surgery to assess the risk of intraoperative hypotension.^71,78^
- Noteworthy studies: Bihorac *et al*.^29^ and Feng *et al*.^41^ created MysurgicalRisk, an ensemble model integrated with their hospital electronic health record system. This system, using more than 285 features, performed well and currently represents the best example of dynamic integration of different types of features such as clinical and socioeconomic data. The system was modeled around a specific electronic health record system and could potentially be adapted to other electronic health records. The second, from Xie *et al*.,^73^ used blood metabolomic profiling to predict the risk of postoperative gastrointestinal failure.

#### Real-time Predictive Models

This category of studies encompasses models specifically designed to predict acute perioperative events in real time, delivering timely alerts to clinicians either during surgery or in the immediate post-operative period with the goal of promptly addressing or even preventing the issue.

- Intraoperative monitoring: Other than one study focusing on prediction of bradycardia, the remainder utilized time-series analysis of intraoperative data to enable real-time trending of vital signs. Intraoperative depth of anesthesia using real-time EEG data,^88^ acute events such as intraoperative hypotension,^90–94,132,133^ postoperative hypertension,^51^ bradycardia,^95^ hypoxemia^97^ and blood product use during caesarean section^81^ were modeled. As an example, Cartailler *et al*.^88^ analyzed continuous EEG readings using a model that recognized abnormal wave patterns to identify suppression bursts.^89^
- Postoperative complications: Two studies analyzed time-series data from wearable devices after surgery to anticipate complications in high-risk patients^40^ or respiratory failure in patients receiving opioids.^86^

### Mortality

Twenty-one studies developed models for prognostic stratification of high-risk patients. Mortality outcomes included models predicting any death, regardless of cause, occurring within a fixed time period after surgery, either inside or outside hospital (usually 30 days or 1 yr), divided by the type of surgery.

- Cardiac surgery^30,115,116,130^
- Major elective surgery: Mortality was assessed postoperatively^27–36,100^ in the surgical intensive care unit^35^ or in the hospital.^28,29,32,36,100^ One study predicted 30-day mortality risk related to myocardial injury in noncardiac surgery patients,^37^ while another developed a natural language processing model using deep learning to analyze medical records and obtain diagnoses directly from notes written by a physician.^27–35,38,100^
- Emergency surgery^26,37,38,110–112^
- Mortality in surgical COVID-19 patients: The COVIDSurg collaborative international panel conducted an international prospective study to develop and validate models that predict postoperative mortality risk in patients with perioperative SARS−CoV-2 infection.^39^

Mortality in perioperative medicine is defined as a rare event (probability less than 5%). Consequently, databases used for mortality may exhibit severe outcome imbalances. Of the 21 studies predicting mortality, 19 were missing other metrics or reported either low sensitivity or low precision. These models had a low F1 score (a measure of model accuracy), indicating a high number of false positives. Using a Random Forest model,Yun *et al*.^35^ did report clinically useful results with an F1 score of 0.84 and sensitivity of 0.90. Castela Forte *et al*.^115^ developed a Super Learner Algorithm (Ensemble model) to predict 5-yr mortality after cardiac surgery, reaching the following values: AUC of 0.81, specificity of 0.70, and sensitivity of 0.69.

### Process

Process outcomes models relate to logistical aspects such as postoperative destination and length of stay.These models are usually linked with other types of outcome such as mortality. Thirteen articles focused on predicting non-clinical outcomes, all using models that stratified high-risk patients.

- Unplanned hospital stay: Most studies predicted unplanned hospital stays after ambulatory or day surgery,^101,107–109^ such as an unplanned overnight stay in the hospital.^107–109^
- Need for intensive care unit stay for more than 24 h^99,100,102–104^
- Readmission and discharge timing: Several studies predicted the risk of hospital readmission within 30 days of surgery,^114^ when patients would be ready for hospital discharge^98,102^ or length of stay after orthopedic surgery.^99,103–105^
- Surgical duration prediction: Gabriel *et al*.^106^ developed a XGB regressor to predict case duration in spinal surgery.These studies used tabular data containing previously mentioned features, with the addition of frailty scores.

### Benchmarks

Forty-four articles used different strategies as comparators of their machine learning model performance (supplementary table 1, https://links.lww.com/ALN/D308). Three main types of benchmarks were identified, comparing models against results obtained from:

(1) Multivariate logistic regression^30,31,36,43,48,54,55,60,62,65,67,69,75,76,78,80,95,106,107,110^
(2) Previously validated scores such as perioperative medicine-related scores (*e.g*.,ASA status, POSSUM, Charlson Comorbidity Index, or National Surgical Quality Improvement Program calculator scores)^30,32,49,61,100,104^ or other scores^58,62^ (*e.g*., Bariclot tool, STOP-BANG score, Mallampati test, various frailty indexes, and the acute kidney injury score)^34,49,58,72,79,82,86,114,121^
(3) Clinical assessment^42,52,88^

Overall, the machine learning models described in these articles outperformed their technical or clinical comparator, with an average increase in AUC and accuracy between 0.2-0.3, except for that of Chen *et al*.^38^ where the ASA score alone, despite a lower AUC, had higher accuracy compared to neural network and logistic regression models.

## Discussion

This systematic review demonstrates the current breadth of applications of artificial intelligence and machine learning models in perioperative medicine for both prediction of perioperative complications and prognostication. Most approaches remain in the early stages of development but are generating promising preliminary results. The substantial increase in machine learning research for perioperative medicine applications is evidenced by the more than 100 articles published in the past decade, incorporating several million patients, with over two-thirds appearing in the last 2 yr.The United States and China, the leading countries in artificial intelligence development, contributed the highest number of publications, followed by South Korea. These findings are consistent with the current use of artificial intelligence in other medical fields such as radiology. We would expect applications to continue to grow rapidly in step with nonmedical usage.^134,135^

Our primary finding, derived from the PROBAST assessment (fig. 3), was that a large proportion of published studies exhibit a high or unclear risk of bias. This suggests that the study design or execution may lead to misleading results. Indeed, most studies were based on retrospective data and used only internal validation. Most studies also presented some form of bias in their selection criteria of the population or the structure of data extraction. This may significantly affect the broader validation of the models generated by these studies. Bias in population selection can arise from a variety of factors such as inadequate representation of diverse patient groups, variations in disease prevalence or treatment methods across different geographical regions, and limitations in data availability. Similarly, issues with the structure of data extraction can result in incomplete or inconsistent data sets, which can, in turn, affect the accuracy and reliability of the models generated.

Some studies, particularly those examining mortality, only reported partial metrics for their models. This lack of comprehensive reporting can lead to overestimated performance metrics and excessive faith in the model’s predictions. Another significant source of bias in the analyzed articles stems from an absence of detailed descriptions regarding calibration. This omission hampers the ability to assess the clinical value of the models, as calibration is essential in determining how accurately the predicted probabilities align with observed outcomes. Together, these factors affect the models’ relevance and reliability in a clinical setting. Our analysis highlights important areas for improvement in future research.

Our second major finding, from the *ad hoc* classification, was the heavy reliance on internal validation that was primarily conducted using limited data sets obtained from single centers. Most studies lacked external validation; this was a significant contributor to the high risk of bias identified. Studies with the lowest risk of bias were those that utilized data collected from multicenter studies or were derived from national databases.^105^ In terms of confirming generalizability and clinical implementation of machine learning models, external validation should be mandatory, ideally performed in different hospitals,^136^ and using separate cohorts of patients to evaluate model performance.^136^ The sharp trajectory of machine learning publications relates to the increasing availability of electronic medical data sets that can be interrogated for patterns and outcomes. Machine learning techniques hold great potential in extracting valuable insights from medical data and aiding decision-making. However, machine learning models trained on such limited data may not adequately capture the heterogeneity and complexity of real-world scenarios. Robustness needs to be confirmed before their widespread adoption, especially as models are generated from data that are not necessarily collected in other institutions. Populations may also differ in crucial respects.

Third, we identified challenges with models predicting perioperative mortality. Mortality rates are now low in elective surgery. Data sets are thus highly imbalanced and can skew predictive models toward exhibiting high false-positive rates. There are several implications arising from such performance issues, as the overestimation of mortality risk could lead to an unnecessary psychologic burden on the patient and a management dilemma for clinicians. Exploring different types of features, such as physiologic variables derived from preoperative tests such as cardiopulmonary exercise testing, or adopting classical approaches may hold the key to improving the accuracy and reliability of mortality prediction models in perioperative medicine. Recent work has suggested that instead of providing incremental value for predicting uncommon outcomes in large data sets, machine learning methods generally do not outperform classical statistical learning methods, which have been found to perform well in low-dimensional settings with large data sets.^137^

These findings demonstrate that use of artificial intelligence and machine learning in perioperative medicine is still in the early stages of development compared to other specialties such as radiology and ophthalmology, *e.g*., for cancer screening and retinopathy.^138,139^ Whereas the use of machine learning in these specialties are primarily used as diagnostic aids, its use in perioperative medicine encompasses a broad range of applications including prognostication, analyzing vital signs for clinical decision support, and predicting complications. The analytical tools and technologies developed for radiology image processing and analysis are generally more robust, well established, and validated. The size, breadth, and quality of large databases in perioperative medicine are limited but improving, and confirmatory external validation is largely lacking. Validated and generalizable machine learning models will provide perioperative medicine clinicians with valuable insights including a wealth of data for inferential research and assistance in decision-making, both for clinical management support and for identifying the appropriate level of postoperative care.

A noticeable trend is the emergence of machine learning models integrated into the hospital electronic healthcare record system such as that described by Bihorac *et al*.^29^ These systems utilize machine learning algorithms and deep learning models to analyze patient data throughout their hospital stay, essentially tracking their clinical journey. The goals are to provide clinicians with objective contemporaneous data to support clinical decisions and to empower patients to make informed decisions. Although promising, their widespread clinical implementation is still distant. Development and deployment of real-time decision support system models are outside the scope of this review but also hold great potential if outcome benefits can be formally and prospectively demonstrated through earlier recognition of deterioration and/ or guided management. For example, it is still unclear whether interventions that reduce the incidence and duration of intraoperative hypotension will ultimately improve patient outcomes.^140^

### Recommendations

While artificial intelligence and machine learning hold great potential in revolutionizing perioperative medicine and improving outcomes, current limitations must be first addressed, such as the issues addressed above regarding bias, external validation, generalizability, and achieving model stability. Other reviews on medical applications of artificial intelligence and machine learning provide more detailed insights.^141–146^

Progress has been made in understanding the limitations of human cognition, but significant gaps still remain.^147^ We therefore recommend adopting a human-centered design approach in conjunction with a continuous artificial intelligence development cycle with the aim of enhancing clinician performance.

To enhance the quality of databases and, subsequently, the models from which they are derived, we propose a multimodal approach that integrates diverse data from various sources, *e.g*., physiologic, biochemical, genetic, and imaging. Many machine learning models are data hungry; to avoid overfitting, integration of diverse data can be a key strategy in developing more robust and reliable models.^148^ The creation and integration of machine learning models into electronic healthcare records can address biases and limitations. However, careful design and quality control are necessary to ensure data utility beyond billing or workflow measurement.

### Study Limitations

It was not possible to objectively assess the data sets of the publications, so we relied upon limitations reported by the authors. Our insights into limitations are also limited by the quality and completeness of the articles. It was not possible to access underlying code or data sets in most publications assessed, nor was it possible to assess validation methods. Last, despite conducting a thorough systematic review, some articles may have been inadvertently over-looked. Nonetheless, the consistency and strength of our findings demonstrate that the trends we have identified are likely to be reflected elsewhere.

## Conclusions and Future Prospectives

The growing complexity and volume of data in perioperative medicine underscore the theoretical potential of artificial intelligence and machine learning in this field. Possible applications could range from risk assessment to real-time treatment guidance. While the development of these technologies could potentially enhance patient care and healthcare resource utilization, the realization of these benefits requires careful consideration of the current limitations and challenges in the field.The potential for early, accurate diagnosis of organ dysfunction or other complications leading to timely or even pre-emptive treatment is an intriguing prospect but must be approached with rigorous validation and proper scrutiny to ensure improved outcomes and resource efficiency.

Significant challenges exist, as highlighted by our review, which revealed important biases and limitations in the current application of machine learning. Until these challenges are overcome, they will impede broad implementation. An overarching strategy is needed to guide the development and application of machine learning.The United Kingdom Department of Health and Social Care issued a code of conduct in 2018, while the U.S. Food and Drug Administration has developed a regulatory framework and action plan. The primary aim of these initiatives is to establish a reliable structure that ensures secure and efficient integration of artificial intelligence and machine learning technologies in the healthcare domain.^96,149–151^ These documents cover aspects such as patient consent for data usage, appropriate handling of data, the need for algorithmic transparency, and accountability. Ethical and legal barriers necessitate structured design and deployment. Since these technologies are intended to assist patients, their future development will necessitate collaboration with policymakers, bioethicists, lawyers, academics, clinicians, patients, and society at large.

### Research Support

Supported by funds from the Cleveland Clinic London Hospital, London, United Kingdom (to Dr. Arina). Supported in part by the Wellcome/EPSRC Center for Interventional and Surgical Sciences at University College London (London, United Kingdom) under grant Nos. 203145Z/16/Z and NS/A000050/1 (to Dr. Mazomenos).

## Supplementary Material

Supplementary Material

Supplementary Information

## Figures and Tables

**Table 1:** *Ad Hoc* Author Classification to Describe the Development Stage toward Clinical Application of the Models Described in the Studies

Classification	Development Stage	Number of Studies
1	Model conceptualization	1
2	Retrospective study, model internal validation	76
3	Retrospective study, model external validation	13
4	Prospective study, nonrandomized	12
5	Prospective study, randomized	1

**Table 2:** Summary of Outcomes and Events in Elective, Emergency, and Cardiac Surgeries

Surgery	Outcomes and Events
Elective noncardiac surgery	
Mortality	30 days and/or 1 yr^26–38^
	In surgical patients with perioperative SARS–CoV-2^39^
Morbidity	Multiple postoperative complications^26,27,29,40–51^
	Acute and chronic pain^52–57^
	Acute kidney failure^52,58–63^
	ASA score prediction^64^
	Delirium and cognitive decline^65–70^
	Cerebral/myocardial infarction^71^
	Difficult intubation prediciton^72^
	Ileus^73^
	Infection risk^74–76^
	Myocardial injury^77^
	Nausea and vomiting^78^
	Obstructive apnoea screening^79^
	Perioperative transfusion^80,81^
	Postoperative atrial fibrillation^82^
	Respiratory failure and depression^43,83–86^
	Sepsis^83,87^
Intraoperative events	EEG interpretation^88^
	Hypotension prediction^89–95^
	Hypoxemia^97^
Process	Length of stay in hospital and ICU^98–105^
	Duration of surgery^106^
	Unplanned overnight admission^107–109^
Emergency noncardiac surgery	
Mortality	30 days^110,111^
Morbidity	Postoperative complications^110,112^
	Respiratory failure^113^
Process	Hospital readmision^114^
Cardiac surgery	
Mortality	30 days, 1 yr, or 5 yr^115,116^
Morbidity	Liver failure^117^
	Major bleeding^118,119^
	Kidney failure^120–122^
	Postoperative complications^123^
Intraoperative events	Hypotension^124^

ASA, American Society of Anesthesiologists; EEG, electroencephalography; ICU, intensive care unit.

## Editor’s Perspective

### What We Already Know about This Topic

- Artificial intelligence and machine learning may offer a novel approach to better predict perioperative outcomes.

### What This Article Tells Us That Is New

- This systematic review and meta-analysis identified 103 studies that employed artificial intelligence or machine learning to predict perioperative outcomes, but the overall quality was only modest with only 13% being externally validated. The authors conclude that the artificial intelligence and machine learning may hold great promise but are not ready for prime time.
