# Supplementary material for: Prediction of Complications and Prognostication in Perioperative Medicine: A Systematic Review and PROBAST Assessment of Machine Learning Tools
Source: Anesthesiology. Author manuscript; Available in PMC 2024 Jun 4. (PMC11146190; doi:10.1097/ALN.0000000000004764)
Supplement: Supplementary Material [file EMS191514-supplement-Supplementary_Material.pdf]

Supplementary Table 1 Summary of data extracted for each paper included in the systematic review with a focus on features, outcomes and limitations stated.

Summary of data extracted for each paper included in the systematic review with a focus on PROBAST topic area, type of study, features, development stage according to authors, machine learning model, metrics and benchmarks. Legend: [*] best performance model in the study, ANN: Artificial Neural network; BB: Balanced Bagging; BERT-DNN: Deep Neural Network model with bidirectional encoder representations from transformers; BRF: Balanced Random Forest; DBN: Deep Belief Network; EEG: Electro-Encephalogram; GAM-NN: Generalized Additive Models with Neural Networks; GBM: Gradient Boosting Machine; GLM: Generalized linear model; GNB: Gaussian Naïve Bayes; ICU: Intensive Care Unit; LDA: Linear Discriminant Analysis; LDC: Linear Discriminant Classifier; Light GBM: Light Gradient Boosting Machine; LinR; Linear Regression; LR: Logistical Regression; MARS: Multivariate Adaptive Regression Splines; MLP: Multilayer Perceptron; ML: Machine Learning; MPCNN-CNN: Multipath Convolutional Neural Network model, Convolutional Neural Network method of handling time-series; MPCNN-LSTM: Multipath Convolutional Neural Network model, long short-term memory method of handling time-series; NB: Naïve Bayes; NN: Neural Network; OLT: Orthotopic liver transplantation; RF: Random Forest; PACU: Post Anaesthesia Care Unit; PTS: patients; RR: Regularized Regression; SVM: Support Vector Machine; XGBoost: eXtreme Gradient Boosting.

SGB: Stochastic Gradient Boosting

Outcome	Author, year, country - Study design, N° of pts	Topic Area	Development (1), Validation (2), both (3) - Diagnostic (D) or Prognostic (P)	Features	Machine Learning & Performance	Validation	Development stage	Benchmark
Cardiac surgery - Mortality	Castela Forte J., 2021, Netherland[110] - Retrospective, single centre, 8241 pts	5-years mortality post cardiac valve and CABG operations	1 - P	Demographics, intra and post-operative vitals and laboratory values	Super Learner (SL) Algorithm[*], ensemble using XGB, BART (Bayesian additive regression trees), LR, SVM, extremely random trees - SL on all data: AUC 0.81, sensitivity specificty, Estimated calibration index (ECI) 0.291, sensitivity = 0.177, specificity = 0.986, cut-off defined by the maximum Youden index: sensitivity = 0.701, specificity = 0.699	Internal validation k-fold-cross-validated	2	NA
Cardiac surgery - Mortality	Fernandes M. P. B., 2021, USA[48] - Retrospective, single centre, 5015 pts	ML to predict 30 days mortality after cardiac surgery	1 - P	Demographics, Echocardiography, renal failure, category of surgery, Cardio-pulmonary bypass (CPB), duration cross clamp-time, vasopressors dose	LR,RF[*], NN, SVM, XGBoost[*] - Best results for 3 different phases. Outside CPB (cardiopulmonary bypass) phase: XGBoost AUC of 0.87, sensitivity 0.75, specificity 0.85, PPV 0.10 - CPB phase: RF AUC of 0.86, Total surgery phase: XGBoost AUC of 0.85	Internal validation with train-test split, 25% of the database used for testing	2	NA
Cardiac surgery - intraop - Morbidity	Li X. F., 2021, China[50] - Retrospective, single centre, 3030 pts	Prediction of hypotension after anaesthesia induction in cardiac surgery	1 - P	Demographics, comorbidities, ASA score, preoperative drugs, type of surgery, EUROscore 1, ECHO, intraoperative values and drugs	RF - RF: AUC of 0.84, specificity 0.85, sensitivity 0.78	Internal validation with Hold-out method, 30% of the database used for testing	2	NA
Cardiac surgery - Morbidity	Gao Y., 2022, China[64] - Retrospective, single centre, 1045 pts	Perioperative major bleeding post cardiac surgery	1 - P	Demographics, type of surgery, clinical and laboratory parameters, antiplatelets use in patients presenting with CAD	LR, SVM, GBT, XGB, RF, Conditional inference random forest (CIRF), boosted classification trees, Naive bayes (NB), classificiation and regression tree CART [*] - CART: AUC=0.831	Internal validation, slit 70/30, five-fold cross validation	2	TRUST score, WILL-BLEED risk score
Cardiac surgery - Morbidity	Jiang H., 2021, China[56] - Retrospective, single centre, 1488 pts	Complication after Mitral valve surgery	1 - P	Demographic characteristics, concomitant disease, preoperative laboratory indicators, operation type, intraoperative information,	XGB[*], SVM, LR, RF, GBM, KNN, NB, AdaBoost - XGB: AUC 0.895, accuracy 0.81, sensitivity 0.89, specificity 0.81, F1-score 0.26	Internal validation, ten-fold cross validaiton	2	NA

Cardiac surgery - Morbidity	Lee H. C., 2018, South Korea[85] - Retrospective, single centre, 2010 pts	Prediction of Acute Kidney Injury after Cardiac Surgery	1 - P	Demographics, comorbidities, preoperative medications and blood values, type of surgery, anaesthesia duration, intraoperative transfusion, intraoperative values, colloid use, type of anaesthesia	DT, RF, GBM[*], SVM, NN, DBN - GBM: AUC 0.78	Internal validation with Hold-out method, 50% of the database used for testing	2	AKICS score, LR
Cardiac surgery - Morbidity	Li T., 2022, China[88] - Retrospective, single centre, 102 pts	Acute Kidney Injury stage 3 after heart transplantation	1 - P	Demographics, past medical history, type of surgery, type of anaesthesia, echography values	LR with L2 regularization - LR: AUC 0.821, sensitivity 0,80, specificity 0.86	Internal validation, 10 cross fold validation	2	Cleveland clinical model
Cardiac surgery - Morbidity	Petrosyan Y., 2022, Canada[89] - Retrospective, single centre, 6522 pts	Prediction of Acute Kidney Injury post cardiac surgery	1 - P	demographics, past medical history, NYHA classification, laboratory data	ensemble model using RF follwed by LR - Ensemble: AUC 0.75, Specificity 0.94, Sensitivity 0.67	Internal validation	2	NA
Cardiac surgery - Morbidity	Shi S., 2021, China[90] - Retrospective, single centre, 672 pts	Prediction of postoperative liver dysfunction after aortic arch surgery	1 - P	Demographics, past medical history, type of surgery, echocardiography, intraoperative fluid and blood use, postoperative liver function	LR, SVM, RF, NB[*] - NB: AUC 0.88	Internal validation, 70/30 random split	2	NA
Cardiac surgery - Morbidity	Tschoellitsch T., 2022, Austria[96] - Retrospective, single centre, 3782 pts	Prediction of massive perioperative allogeneic blood transfusion in cardiac surgery	1 - P	Hospital admission, past medical history,	RF, NN, GBM, ADA (adaptive boosting), LR - RF: AUC 0.81, F1-score 0.15, NPV 0.987, PPV 0.110	Internal validation, five-fold cross validation	2	NA
Cardiac surgery - Mortality	Castela Forte J., 2022, Netherland[111] - Retrospective, single centre, 9415 pts	Comparison of perioperative feature use to predict overall mortality rates at 30 days, 1 year, and 5 years in cardiac surgery	2 - P	Demographics, intra and post-operative vitals and laboratory values	LSTM (long short term memory) NN [*], postoperative data analysis with XGB - LSTM (30 d mortality with full perioperative): AUC 0.81, Sensistivity 0.59, Specificity 0.82 - LSTM (1 year mortality with full perioperative): AUC 0.82, Sensitivity 0.54, Specificity 0.88	Internal validation, 10-fold cross-validation	2	NA
Cesarean delivery - Morbidity	Ren W., 2022, China[95] - Retrospective, single centre, 600 pts	Predicting the amount of blood transfusion during caesarean section and analysing the risk factors of hypothermia during anaesthesia recovery	1 - P	Demographics, diagnosis, admission time, type of anaesthesia	LR, XGBoost, ANN[*] - ANN: AUC 0.957, F1-score 0.951, recall 0.955	Internal validation with Hold-out method, 25% of the database used for testing	2	NA
Emergency surgery - Morbidity	Xue Q., 2021, China[65] - Retrospective, single centre, 926 pts, emergency surgery	Predict pulmonary complications after emergency gastrointestinal surgery	1 - P	Laboratory values, past medical history, demographics, location of lesion	LR[*], XGB, Decision tree, gradient boosting, - LR: AUC 0.836	Internal validation, Five-fold cross-validation	2	NA

Emergency surgery - Morbidity and Mortality	Bertsimas D., 2018, USA[63] - Retrospective, multiple centres from American College of Surgeons (ACS) National Surgical Quality Improvement Program (ACS-NSQIP) database, 382960 pats, emergency patients	Design an interactive, nonlinear risk calculator for Emergency Surgery (ES) to predict 30 days mortality and morbidity	1 - P	From ACS-NSQIP database, patient demographics, comorbidities, laboratory variables, and wound characteristics	Optimal classification trees (OCT) - Mortality AUC 0.92	Internal validation with Hold-out method	2	LR
Emergency surgery - Morbidity and Mortality	El Hechi M.W, 2021, USA[39] - Retrospective, multiple centres, 59955 pts, emergency patients	Validarion of Predictive Optimal Trees in Emergency Surgery Risk (POTTER) tool to predict 30 days mortality and morbidity after emergency surgery	2 - P	From ACS-NSQIP database, patient demographics, comorbidities, laboratory variables	Optimal classification trees (OCT) (see Bertimas, D, 2018) - Mortality AUC 0.93	External validation	3	NA
Emergency surgery - Mortality	Gao J., 2021, USA[122] - Retrospective, multicentre using the national surgical outcome database (National Surgical Quality Improvement Program - NSQIP), 177872 pts, emergency surgery	Mortality 30 days following Emergency General Surgery	2 - P	From ACS-NSQIP database, demographics, past medical history, steroids use, type of surgery	GBM, NN (ANN), ensemble voting classifier (VC)[*] - VC: AUC 0.956, sensitivity 0.948, specificity 0.855, PPV 0.214, NPV 0.997	Internal validation, 10-fold stratified cross-validation, split 90/10	2	NA
Emergency surgery - Procedural	Misic V., 2020, USA[42] - Retrospective, multi centre, 34532 pts, emergency surgery	30 days readmission via the emergency department	3 - P	Demographics, past medical history, ASA score, surgical blood loss, tracheostomy, mechanical ventilation, readmission to hospital	LR, GBDT[*], RF - GBDT (general + labs + meds + teams + cpt): AUC=0.867, Brier score=0.045	External validation with external database and temporal validation	3	HOSPITAL and LACE scores
Liver transplant - Morbidity	Chen C, 2023, China[91] - Retrospective, single centre, 786 pts	Postoperative sepsis post liver trasplant	3 - P	Meld Score, child pugh socre	LR, SVM, GNB, RF[*], GBM - RF, AUC 0.731, accuracy 0.71, sensitivity 0.62, specificity 0.76	External validation with an independent dataset from same hospital	3	NA
Liver transplant - Morbidity	Chen C., 2021, China[37] - Retrospective, single centre, 591 pts	Predict pneumonia after Orthotopic liver transplantation	1 - P	Demographics, Preoperative comorbidities, aetiology of liver failure, perioperative laboratory values, preoperative complications, microorganism observation, intraoperative medications, fluids and transfusion, postoperative medication.	XGBoost[*], LR, SVM, RF, MLP, GBM - XGBoost: AUC 0.79, sensitivity 0.52, specificity 0.77	Internal validation with hold-out method, 30% of the database used for testing	2	NA
Liver transplant - Morbidity	Kamaleswaran R., 2021, USA[92] - Retrospective, single centre, 5748 pts	Ealry sepsis after liver trasplant	1 - P	3-h observational window continuous for six physiological data streams [heart rate, respiratory rate, oxygen saturation, systolic blood pressure (SBP), diastolic blood pressure (DBP), and mean femoral artery blood pressure (MAP)	XGB[*], LR, SVM, RF, - XGB: AUC 0.97, Precision-recall 0.97	Internal validation, 5-fold cross validation	2	NA

Liver transplant - Morbidity	Lee H. C., 2018, South Korea[86] - Retrospective, single centre, 1211 pts	Prediction of Acute Kidney Injury after liver transplant	1 - P	Demographics, comorbidities, preoperative medications, cause of liver transplant, preoperative status (Child-Turcotte-Pugh score), preoperative blood measurements, surgery type and anaesthesia details, intraoperative transfusions, drugs and measurements	DT, RF, GBM[*], SVM, NB, MLP, DBN, LR - GBM: AUC 0.90, accuracy 0.84	Internal validation with Hold-out method, 30% of the database used for testing	2	LR
Liver transplant - Morbidity	Zhang Y., 2021, China[28] - Retrospective, single centre, 780 pts	Prediction of acute kidney injury (AKI) after liver transplantation (LT)	2 - P	Demographics, comorbidities, aetiology of liver and complications, preoperative values, intraoperative incidents, medications, fluid infusion and blood transfusion, postoperative medications	LR, SVM, RF, GBM[*], AdaBoost - GBM: AUC 0.76, F1-score 0.73	External validation with independent Database involving 195 cases. Internal validation with hold-out method, 30% of the database used for testing	3	AKI prediction score
Major elective non-Cardiac surgery - Morbidity	Jeong Y. S., 2021, South Korea[55] - Retrospective, single centre, 3220 pts	Predict postoperative complication and major adverse cardiac events in patients with End Stage Renal Disease	1 - P	Demographics, perioperative blood values, type of anaesthesia, type of surgery	SVM, DT, RF, GNB, ANN, LR[*], XGBoost - RF with demographic, electrolyte, anaesthesia and operation features: F1 Score of 0.796. AUC not reported, NLP used for feature estraction.	Internal validation with hold-out method, 53 pts used for testing	2	NA
Major elective non-Cardiac surgery - Intraop - Morbidity	Adhikari L., 2019, USA[87] - Retrospective, single centre, 2911 pts	Prediction of acute kidney injury with intraoperative data	1 - P	69 preoperative variables, intraoperative time-series features	IDEA (Intraoperative Data Embedded Analytics) algorithm based on generalized additive model (GAM) with logistic link function and RF - For 7 days AKI: AUC 0.86, accuracy 0.80, sensitivity 0.81, PPV 0.74, NPV 0.85	Internal validation with randomly split into 70% for training and 30% for validation	2	NA
Major elective non-Cardiac surgery - Intraop - Morbidity	Cartailler J., 2019, France[100] - Prospective, single centre, 79 pts	Prediction of Iso-electric suppressions from EEG during anaesthesia using a Machine Learning Model	1 - P	Demographics, intraoperativecontinuous EEG features, perioperative vitals (MAP)	Univariate LR and multivariate LR[*] - Univariate LR: AUC of 0.90, multivariate LR: AUC of 0.93	Method of validation not stated	4	NA
Major elective non-Cardiac surgery - intraop - Morbidity	Hatib F., 2018, USA[27] - Retrospective, single centre + Prospective, cohort, single centre, 1334 pts retrospective study + 204 pts prospective study	Prediction of intraoperative hypotension	2 - P	Arterial waveform features	LR with patented preprocessing pipeline - NA	Internal validation	2+4	NA
Major elective non-Cardiac surgery - intraop - Morbidity	Jacquet-Lagreze M., 2019, France[101] - Retrospective, multicentre, 83 Pts	Build a predictive model of intraoperative hypotension using linear extrapolation (LepMAP)	1 - P	10 minutes segments of intraoperative arterial pressure analysis	Linear extrapolation (LepMAP) - 1 minute before occurance - LepMAP: AUC of 0.97, 2 minute before occurance - LepMAP: AUC of 0.81 , 5 minute before occurance - LepMAP: AUC of 0.69	Internal validation, 2000 repetitions of bootstrapping	2	NA

Major elective non-Cardiac surgery - intraop - Morbidity	Kang A. R., 2020, South Korea[102] - Retrospective, single centre, 222 Pts	Predict the risk of intraoperative hypotension post anaesthesia induction	1 - P	Demographics, ASA score, comorbidities, intraoperative vitals, Standardized anaesthesia induction (TCI with propofol and remifentanil), intraoperative vital signs, mechanical ventilation, pharmacological data, vasoactive drug administration in laparoscopic cholecystectomy	NB, LR, RF[*], ANN - RF: AUC of 0.8423, precision: 0.81, accuracy: 0.79	internal validation with K-fold cross-validation, number of folds not stated	2	NA
Major elective non-Cardiac surgery - Intraop - Morbidity	Kendale S., 2018, USA[106] - Retrospective, single centre, 13323 pts	Prediction of postinduction hypotension in patient requiring general anaesthesia	1 - P	Demographic, ASA score, comorbidities, Preoperative medications, type of surgery, intraoperative medications, ventilatory settings, intraoperative vitals	LR, SVM, NB, KNN, LDA, RF, NN, GBM[*] - GBM: AUC 0.76, sensitivity 0.64, Specifity 0.75	Internal validation with Hold-out method, 30% of the database used for testing	2	NA
Major elective non-Cardiac surgery - Intraop - Morbidity	Lee S., 2021, South Korea[105] - Retrospective, single centre, 3301 pts	Real-time predictions 5, 10, and 15 min before a hypotensive event	1 - P	Continous data from invasive and non invasive arterial pressure waveform and electrocardiography, photoplethysmography, capnography	NN Binary classifier model[*], LR model - 5 min prediction arterial pressure only model NN: AUROC 0.931, sensitivity 0.85, specificity 0.85	Method of validation not stated	2	LR
Major elective non-Cardiac surgery - intraop - Morbidity	Lundberg S. M., 2018, USA[108] - Retrospective, single centre, 48069 pts	Predict hypoxemia during surgery using a ML based model (Prescience)	1 - P	Demographics, surgery type, preoperative medication, time-series intraoperative vitals (saturation)	GBM - AUC of 0.92	Internal validation with Hold-out method, 963674 samples used for model testing	2	Anaesthesiologist's ability to predict hypoxemia
Major elective non-Cardiac surgery - Intraop - Morbidity	Maheshwari K., 2020, USA[104] - Prospective, single centre, 320 pts	Predict hypotension with non-invasive arterial pressure waveforms	1 - P	Demographics, past medical history, Intraoperative waveform	Ad hoc model described in Hatib et al - HPI: AUC of 0.93, sensitivity 0.86, specificity 0.86	Method of validation not stated	4	NA
Major elective non-Cardiac surgery - intraop - Morbidity	Schenk J., 2021, Netherlands[103] - Sub-study of prospective trial (Hypotension prediction trial - HYPE), 60 pts	Assessment of the use of intraoperative Hypotension Predictor Index (HPI) guided haemodynamic care to prediction development of postoperative hypotension	1 - P	Demographics, comorbidities, pre-procedure medication, type of surgery, intraoperative medication and fluid balance, duration of anaesthesia, postoperative monitoring, type of anaesthesia, postoperative fluid balance, postoperative mechanical ventilation and its duration, postoperative complications	Ad hoc model described in Hatib et al - Intraoperative HPI-guided haemodynamic care did reduce IOH, but did not lower the Time-weight average of POH.	Method of validation not stated	4	NA
Major elective non-Cardiac surgery - intraop - Morbidity	Solomon S. C., 2020, USA[107] - Retrospective, single centre, 62182 pts	Predicting the occurrence of intraoperative bradycardia	1 - P	Demographics, ASA score, medical history, preoperative vital signs, medication history, operating room location, procedure type, anaesthesia type, intraoperative vital sign data, all administered bolus and infused medications, fluid administration, ventilator data including respiratory rate and tidal volume	GBM - Predicted at 3 time points, induction of anaesthesia - AUC of 0.81, start of the procedure - AUC of 0.87, 30 minutes after the start of the procedure - AUC of 0.89.	Internal validation with 10-fold cross-validation	2	LR

Major elective non-Cardiac surgery - intraop - Morbidity	Wijnberge M., 2020, Netherlands[35] - Prospective, single centre, 68 pts	Investigating the use of a early warning system for intraoperative hypotension.	1 - P	Demographics, ASA score, WHO classification, type of surgery, surgical approach, intraoperative values	Ad hoc model described in Hatib et al - ML model application significantly reduced the time-weighted average of hypotension during surgery	Method of validation not stated	5	Comparison with standard care treatment
Major elective non-Cardiac surgery - Morbidity	Awadalla S. S., 2022, USA[67] - Retrospective, cohort, single centre, 2106 pts	Clinically meaningful pain (>3 on a 0–10 scale) at 30-days and 1-year after surgery were the primary and secondary outcomes	1 - P	Patient response to surveys, demography, ASA, Charles Score, surgical category, anaesthesia Type	k-means clustering, LR - Clusterization was significant on meaningful postsurgical pain at 30 days (p<0.001), but not at 1-year after surgery (p=0.79). Cluster IV: AUC of 0.087	Hosmer-Lemeshow goodness-of-fit test, internal validation with cross-validation performed	2	NA
Major elective non-Cardiac surgery - Morbidity	Bishara A., 2021, USA[81] - Retrospective, single centre, 29004 pts	OPAL - Post-op Acute Kidney Injury development	1 - P	Demographics, ASA score, Type of surgery	k-means clustering, GBM [*], LR - GBM: AUC of 0.85, Sensibility 0.9, Specificity 0.8	Internal hold-out method, 20% of the database used for testing	2	LR
Major elective non-Cardiac surgery - Morbidity	Bishara A., 2022, USA[76] - Retrospective, single centre, 24885 pts	Postoperative delirium detection	1 - P	Demographics, ASA score, in total 115 predictor variables, type of surgery, emergency surgery	LR based on features selected by clinicians, LR based on machine learning feature selection, XGBoost [*], NN - NN: AUC 0.84, sensitivity 0.72, specificity 0.77	Internal 10-fold cross-validation	2	Two LR models (‘ML hybrid’ and ‘clinician guided’), delirium risk stratifcation tool (AWOL-S)
Major elective non-Cardiac surgery - Morbidity	Bolourani S., 2020, USA[83] - Retrospective, multiple centres, 4062	Respitatory failure post pulmonary lobectomy	1 - P	From National Inpatient Sample (NIS), patient demographics, past medical history, type of surgery, intraoperative events,	RF with and without SMOTE - RF Sensitivity 0.75,Specificity 0.99, AUC 0.83 - RF with SMOTE Sensitivity 0.83 Specifitity 0.94, AUC 0.90	Internal validation	2	NA
Major elective non-Cardiac surgery - Morbidity	Brennan M., 2019, USA[33] - Prospective, non-randomized pilot, single centre, 150 pts	Compare the usability and accuracy of preoperative risk assessment between physicians and MySurgeryRisk	1 - P	286 preoperative features, demographics, socio-economics, administrative, clinical, pharmacy, and laboratory variables	MySurgeryRisk model (Zeng, F) - AUC range 0.73 and 0.85	Method of validation not stated	4	Physicians’ Initial Risk Assessement
Major elective non-Cardiac surgery - Morbidity	Chelazzi C., 2021, Italy[54] - Retrospective, single centre, 560 pts	Implementing an automated surgical risk calculator based on Artificial Neural Network technology (SUMPOT) to identify pts at risk for postoperative complications	1 - P	Demographics, ASA score, type of surgery, surgical factors, Clavie-Dindo Score	ANN using Single Layer Feedforward Network (SLFN)[*], BDT - SLFN: AUC of 0.89, sensitivity 0.89, specificity 0.90	Internal cross-validation	2	Binary Decision Tree (AUC 0.58)
Major elective non-Cardiac surgery - Morbidity	Du Y., 2022, China[94] - Retrospective, single centre, 130 pts	Infection risk prediction in postoperative patients with non-mechanical ventilation and intravenous neurotargeted drugs	1 - P	Demographics, opiods use, laboratory data	RF, SVM-RFE (Support Vector Machine-Recursive Feature Elimination)[*], LR, NN - SVM: AUC 0.830	Internal validation	2	LR

Major elective non-Cardiac surgery - Morbidity	Hu X. Y., 2021, China[77] - Secondary analysis of observational study, 531 pts	Create a fully automated score based on ML to predict the incidence of Postoperative delirium	1 - P	Demographics, Charlson comorbidity index, postoperative neutrophil-to-lymphocyte ratio, intraoperative blood loss, anaesthesia duration, extubating time, Intensive care unit admission, mini-mental state examination score (MMSE).	LR[*], RF, XGBoost, SVM - LR: AUC of 0.804, F1-score:0.567	Internal validation with train-test split, 30% of the database used for testing	2	NA
Major elective non-Cardiac surgery - Morbidity	Jones R. N., 2021, USA[79] - Secondary analysis of observational study, 560 pts	Prediction of cognitive decline from 2 to 36 months following elective surgery in a population with age > 70 years	1 - P	Demographics, ASA score, perioperative blood values, perioperative vitals, type of surgery	RR, MARS[*], RF, KNN, GBM, NN, LinR, LR - MARS: AUC of 0.76	Internal validation with hold-out method, 20% of the database used for testing	2	NA
Major elective non-Cardiac surgery - Morbidity	Jungquist C. R., 2019, USA[109] - Prospective, single centre, 48 pts	Predict opioid-induced respiratory depression in postoperative using electronic monitoring devices	1 - P	Patient STOP-BANG questionnaire, length of surgery, postoperative measurement with electronic device measuring: pulse oximetry, Capnography, Minute Ventilation	SVM - Detection rate > 0.9 over short horizons of event (< 5 minutes).	Method of validation not stated	4	STOP-BANG questionnaire
Major elective non-Cardiac surgery - Morbidity	Karri R., 2021, Australia[72] - Retrospective use of MIMIC-III database (national database but single centre based), 6040 pts	Prediction of postoperative atrial fibrillation (POAF) in ICU admitted pts	1 - P	Demographic, comorbidities, admission type	RF, DT, LR, KNN, SVM, GBM[*] - GBM: AUC of 0.74 (0.71–0.77), Sensitivity: 0.60, Specificity: 0.65	Internal validatio with five-fold cross-validation repeated 4 times	2	Comparation with the POAF score, AUC was 0.63
Major elective non-Cardiac surgery - Morbidity	Koretsky M., 2022, USA[52] - Retrospective, multicentre, 6002257	develop scoring systems for the prediction of combined, early, and late postoperative intubation	1 - P	Data from ACS-NSQIP, demographics, past medical hystory, type of surgery	LR, RF, GBM[*] - GBM: AUC 0.9037, Brier score 0.1226	Internal validation, cross validation	2	NA
Major elective non-Cardiac surgery - Morbidity	Kristinsson ÆÖ, 2022, Denmark[26] - Prospective, observational, multi centre, 292 pts	Use of continuous vital signal monitoring to predict postoperative outcomes	1 - P	Demographics, continuous postoperative vitals	KNN, DT, RF[*], BE - RF: AUC 0.65, Sensitivity 0.92, Specificity 0.36, F1-score 0.64	Internal validation with 5-fold cross-validation	4	NA
Major elective non-Cardiac surgery - Morbidity	Li P., 2023, China[93] - Observational, cohort study, 2014 pts	Postoperative infection in elderly	1 - P	Demographics, past medical history, type of surgery, type of anaesthesia, ASA score	NN[*], LR - NN: AUC 0.763, sensitivity 0.632, specificity 0.805	Internal validation, 75/25	2	LR
Major elective non-Cardiac surgery - Morbidity	Mascarella M. A., 2022, Canada[60] - Retrospective, a cohort of the national surgical outcome database from ACS- NSQIP, 43701 pts	Prediction of Major postoperative adverse events (MPAEs) in a perioperative head and neck surgery population	1 - P	Demographics, comorbidities, preoperative functional activity and drugs, ASA Score, type of surgery, wound classification, surgical time, tracheostomy	LR[*], SVM, NN, RF - LR: AUC 0.85	Internal validation with Hold-out method, no percentage of database used for testing stated	2	Modified frailty index 5, ASA score
Major elective non-Cardiac surgery - Morbidity	Nudel J., 2021, USA[59] - Retrospective, cohort based on data from 2015-2017 Metabolic and Bariatric Surgery, Accreditation and Quality Improvement Program database, 436807 pts	Prediction of anastomotic leak and Venous Thromboembolism after bariatric surgery	1 - P	Demographics, comorbidities, ASA score, preoperative blood values, year of surgery, training level of the first assistant,	ANN[*], XGBoost[*], LR - Predicting leak - ANN: AUC 0.75 - VTE - XGBoost: AUC 0.67	Internal validation with Hold-out method, 25% of the database used for testing	2	LR

Major elective non-Cardiac surgery - Morbidity	Oh A., 2023, South Korea[74] - Retrospective, single centre, 6811 pts	Myocardial injury after non-cardiac surgery (MINS) prediction after non cardiac surgery	1 - P	Demographics, past medical history, perioeprative organ support, medication, cardiac troponin	XGB - XGB: AUC 0.74, accuracy 0.79, sensitivity 0.29, specificity 0.93	Internal validation, five-fold cross-validation	2	NA
Major elective non-Cardiac surgery - Morbidity	Peng X., 2022, China[75] - Prospective, single centre, 5705 pts	Postoperative major adverse cardiovascular events (MACEs) in geriatric patients	1 - P	NYHA classification, laboratory data, demographics, past medical history	RF, GBM, XGB[*], SVM, LR - XGB: AUC 0.870, AUPRC 0.404, Brier Score 0.024	Internal validation	4	NA
Major elective non-Cardiac surgery - Morbidity	Racine A. M., 2020, USA[80] - Prospective, single centre, 560 pts	Predict post operative delirium in elder population	1 - P	Demographics, preoperative Laboratory results, surgical procedure, anaesthesia type and duration, inter and post operative medications	LR, GBM, NN[*], RF, RR, Stepwise LR - NN: AUC 0.71, Specificity 0.82, Sensibility 0.50	Internal validation with Hold-out method, 111 pts used for testing	4	Stepwise LR
Major elective non-Cardiac surgery - Morbidity	Sobrie O., 2016, Belgium[49] - Retrospective, multicentre, 898 pts	Proposing a multiple criteria decision aiding system to predict the patient's ASA score and the decision of accepting the patient for a surgery.	1 - P	ASA score, demographics, preoperative blood values, comorbidities, preoperative vital signs, cerebrovascular accident, myocardial infarction	Majority rule sorting model (multiple criteria sorting methods, a simplified version of ELECTRE Tri, an outranking sorting procedure) - Predicting ASA score - AUC of 0.98, predicting the decision of accepting the patient for a surgery - AUC of 0.75	Internal validation with cross-validation performed with 3 different splits	2	SVM, C4.5, KNN, MLP, RBF, and Majority rule voting
Major elective non-Cardiac surgery - Morbidity	Song Y., 2023, China[82] - Retrospective, single centre, 29756 pts	Development of postoperative delirium in elderly patients	1 - P	Demographics, past medical history, ASA score, type of surgery, type of anaesthesia, blood loss	LR[*], RF, GBM, Adaboost, XGB, ensemble model - LR: AUC 0.783, Sensitivity 0.74, specificity 0.70	Internal validation, 70/30 random split	2	NA
Major elective non-Cardiac surgery - Morbidity	Suhre W., 2020, USA[31] - Retrospective, multicentre, 27388 pts	Identifying association of cannabis use with the risk of postoperative nausea and vomiting.	2 - P	Demographics, ASA score, medical history, procedure duration, exposition to Nitrous Oxide, surgery risk for nausea, total number of prophylactic agents, PACU opioids, Apfel score	Bayesian Additive Regression Trees - Daily cannabis use: relative risk for postoperative nausea and vomiting was 1.19 (95 CI% 1.00– 1.45), absolute marginal increase in risk was 3.3% (95% CI 0.4–6.4%). Current, non-daily use of cannabis: relative risk was 1.07, 95% CI 0.94–1.21).	External validation with 16245 pts	3	Comparison with Bayesian logistic regression model
Major elective non-Cardiac surgery - Morbidity	Tavolara T. E., 2021, USA[34] - Prospective, single centre, 152 pts	Predict difficult intubation from frontal face images using deep learning models	1 - P	11 facial regions, 68 landmarks of the face	11 CNN and 11 attention-based MIL making up a robust facial feature extractor (FRFE) - AUC 0.711, sensitivity 0.73, specificity 0.68	Internal validation with Hold-out method, 10% of the database used for testing	4	bedside Mallampati test and thyromental distance test
Major elective non-Cardiac surgery - Morbidity	Tourani R., 2019, USA[32] - Retrospective, multicentre using the national surgical outcome database (National Surgical Quality Improvement Program - NSQIP), 47089 pts	Predicting seven infectious post-surgical complications in 30 days post surgery.	3 - P	Demographics, history of complications, laboratory results and vitals, medications, high-resolution vitals and labs.	LR - Using only demographics and preoperative data - AUC of 0.560 - 0.877. Demographics, preoperative data and intraoperative data - AUC of 0.563 - 0.881	External validation - Model performance was compared between two different clinics - Bootstrap estimation with 200 replications	3	NA

Major elective non-Cardiac surgery - Morbidity	Walczak S., 2020, USA[44] - Retrospective, multi centre, 1600000 pts	Prediction of operative transfusion	1 - P	Data from ACS-NSQIP, demographics, past medical hystory, type of surgery	ANN - AUC 0.858, sensitivity 0.75, specifity 0.70	External validation with indipendent dataset	3	LR
Major elective non-Cardiac surgery - Morbidity	Xie T., 2021, China[99] - Prospective, single centre, 48 pts	Predicting postoperative recovery of gastrointestinal function based on metabolomic profiling. Indicating potential mechanisms contributing to gastrointestinal function after surgical resection of colorectal cancer under the fluid management of goal-directed fluid therapy.	1 - P	Perioperative metabolomics	SVM, DT, RF, GNB, ANN, LR[*], XGBoost - LR with 22 features: AUC 0.98, accuracy of 0.92	Human validated	4	NA
Major elective non-Cardiac surgery - Morbidity	Xue B., 2021, USA[57] - Retrospcetive, single centre, 111888 pts	Predicting the risk of postoperative complications related to pneumonia, acute kidney injury, deep vein thrombosis, pulmonary embolism, and delirium	1 - P	Patient and clinical characteristics available preoperatively, intraoperatively, and a combination of both	LR, SVM, RF, GBT[*], DNN - GBT: AUC 0.905, AUPRC 0.208, sensitivity 0.525, specificity 0.95, F1-score 0.277, accuracy 0.941	Internal validation with 5 random shuffles of 5-fold cross-validation	2	NA
Major elective non-Cardiac surgery - Morbidity	Zhang L., 2021, China[51] - Cross-sectional study om NSQIP database, 481 pts	Screening of obstructive sleep apnoea (OSA) in a perioperative population using facio-cervical measurements	1 - P	Demographics, Faciocervical measurements	SVM - SVM: AUC 0.83, sensitivity 0.89, specificity 0.76	Internal validation with 10-fold cross-validation	2	STOP-BANG questionnaire
Major elective non-Cardiac surgery - Morbidity and Mortality	Bihorac A., 2019, USA[98] - Retrospective, single centre, 51457 pts	Postoperative complications (acute kidney injury, sepsis, venous thromboembolism, intensive care unit admission > 48 hours, mechanical ventilation > 48 h, wound, neurologic, cardiovascular complications) and death at 1-, 3-, 6-, 12-, and 24-months	1 - P	285 preoperative features, demographics, socio-economics, administrative, clinical, pharmacy, and laboratory variables	MySurgicalRisk model (Feng Z) - AUC ranging between 0.82 and 0.94, sensitivity 0.74, specificity 0.69, death at 1-, 3-, 6-, 12-, and 24-month - AUC ranging between 0.77 and 0.83	Internal 50-time repeated 5-fold cross-validation runs	2	NA
Major elective non-Cardiac surgery - Morbidity and Mortality	Bonde A., 2021, USA[36] - Retrospective, multiple centre from American College of Surgeons (ACS) National Surgical Quality Improvement Program database, 5881881 pts, emergency patients	Predicting mortality and 18 different morbidity postsurgery	3 - P	From ACS - NSQIP, 58 features, demographic, laboratory values	3 NN, of increased complexity. The three models were developed with 1 134 080, 1 186 327, and 1 264 846 trainable variables and 21, 35, and 57 embedding layers, respectively. - The mean AUCs for the validation set were 0·864 for model 1, 0·871 for model 2, and 0·882 for model 3. The mean AUCs for the test set were 0·859 for model 1, 0·863 for model 2, and 0·874 for model 3	External validation with patients who were treated at a large US hospital, excluded them from the base dataset, and reserved this independent group for final model testing. Internal validation with a training set and a validation set from the remaining cases	3	NA

Major elective non-Cardiac surgery - Morbidity and Mortality	Chae D., 2022, South Korea[61] - Retrospective, single centre, 21510 pts	To develop a risk scoring system for 1-week and 1-month mortality after major non-cardiac surgery, and assess the impact of postoperative factors on 1-week and 1-month mortality	1 - P	Demographics, ASA, preoperative laboratory values, anaesthesia duration and type, intraoperative and postoperative laboratory values, transfusion, urine output, surgery type	penalized regression, RF, SVM, GBM, LightGBM [*] - (Pre- , intra- , and postoperative features) LightGBM: AUC 0.90, Brier score 0.014	Internal cross-validation	2	NA
Major elective non-Cardiac surgery - Morbidity and Mortality	Feng Z., 2017, USA[29] - Proposal for Intelligent Perioperative System (IPS) for real-time assessment of risk of postoperative complications	Proposal for Intelligent Perioperative System (IPS) for real-time assessment of risk of postoperative complications	- P	NA	Preliminary description of ML model (MySurgeryRisk) based on the Generative Additive Model (GAM). - NA	Method of validation not stated	1	NA
Major elective non-Cardiac surgery - Morbidity and Mortality	Hofer I.S., 2020, USA[53] - Retrospective, single centre, 59981 pts	Predict postoperative mortality, acute kidney injury, and reintubation	1 - P	46 features available at the end of the surgery, including drug dosing, blood loss, vital signs, and others were extracted, six additional features accounting for total intraoperative hypotension	DNN - DNN: AUC 0.792 for AKI, 0.879 for reintubation, 0.907 for mortality, and 0.874 for any outcome. Average F1-score 0.47	Internal validation 80/20	2	LR, ASA, risk stratification index, risk quantification index
Major elective non-Cardiac surgery - Morbidity and Mortality	MacKay E., 2021, USA[41] - Retrospective, cohort study, 38539 pts	Primary outcome was 30-day mortality. Secondary outcomes were: rehospitalization, and any of 23 adverse clinical events occurring within 30 days of the index admission date.	1 - P	Feature from patients extracted from a random 5% sample from Medicare beneficiaries encompassing fee-for-service (FFS) claims data–Parts A/B claims from 2008–2011	SVM, RF, NN, XGB[*], LR - XGB Mortality: AUC 0.73, Brier Score 0.07, XGB Morbidity: AUC 0.78, Brier Score 0.01	External validation with second indipendent validation dataset	3	NA
Major elective non-Cardiac surgery - Mortality	Chen P.F., 2022, Taiwan [121] - Retrospective, single centre, 121313 pts	To develop a fusion natural language model containing structured and unstructured (text) features to predict the in-hospital 30-day postoperative mortality before surgery, using preoperative diagnosis and the planned procedure.	3 - P	Demographics, comorbidities, preoperative laboratory data, preoperative vital signs, ASA score, type of surgery	BERT-DNN[*], DNN, RF, XGBoost, LR - BERT-DNN: AUC 0.96, AUPRC 0.31	Internal validation with hold-out method. 16 267 samples were used for testing	2	American Society of Anesthesiologist Physical Status.
Major elective non-Cardiac surgery - Mortality	COVIDSurg Collaborative, 2021 ,international panel[123] - Prospective, international, prospective, cohort study, 8492 pts	To develop and validate a machine learning based risk score to predict postoperative mortality risk in patients with perioperative SARS-CoV-2 infection	3 - P	Demographics, preoperative respiratory support, ASA score, revised cardiac risk index (RCRI) score.	RF, DT, GLM, LR[*] - 26 models using different combinations of five selected features - LR with 4 features: AUC 0.80	External validation using Time-based database split for validation	4	PROBAST assessment
Major elective non-Cardiac surgery - Mortality	Fritz B. A., 2019, USA[113] - Retrospective, single centre, 95907 pts	Real time prediction of postoperative 30 days mortality using neural network models	1 - P	Demographics, comorbidities, preoperative vital signs, preoperative laboratory values, intraoperative vital signs, intraoperative ventilator parameters, medications and fluids	MPCNN-LSTM[*], MPCNN-CNN, DNN, RF, SVM, LR - MPCNN-LSTM: AUC of 0.867, AUPRC: 0.095, MPCNN-CNN: AUC of 0.855, DNN: AUC of 0.825, RF: AUC of 0.848, SVM: AUC of 0.836, LR: AUC of 0.837	Method of validation not stated	2	LR
Major elective non-Cardiac surgery - Mortality	Hill B. L., 2019, USA[115] - Retrospective, single centre, 53097 pts	Prediction of mortality using data available at the time of surgery	1 - P	Demographics, comorbidities, perioperative blood analysis and vitals, ASA, type of anaesthesia, type of surgery	LR, ElasticNet LR, RF[*], XGBoost - RF: AUC: 0.936, F1-score: 0.275, Precision 0.610, Specificity:0.998	Internal validation with five-fold cross-validation	2	POSSUM, ASA, Charlson comorbidity scores

Major elective non-Cardiac surgery - Mortality	Lee C. K., 2021, USA[119] - Retrospective, single centre, 59985 pts	Use of interpretable neural network to predict postoperative in-hospital mortality	1 - P	Demographics, ASA score, type of anaesthesia, invasive anaesthesia procedure (arterial line, etc), type of surgery, medication and vitals post-surgery	GAM-NN[*], LR - With Healthcare Cost and Utilization Project (HCUP) features - GAM-NN: AUC of 0.92. Without HCUP features: GAM-NN: AUC of 0.91	Internal validation with hold-out validation method, 11997 pts used for testing	2	With HCUP LR: AUC of 0.91, without HCUP features LR: AUC of 0.906
Major elective non-Cardiac surgery - Mortality	Lee SW, 2022, South Korea[112] - Retrospective, Multiple Centres, 454404 pts, elective and emergency surgery	30 days mortality after a non-cardiac surgery	3 - P	Preoperative medications, medical comorbidities, laboratory data	LG, RF, XGB[*], NN - XGB: AUROC=0.942, AUPRC=0.175	External validation, bootstrapping and grid search with tenfold cross-validation were performed	3	NA
Major elective non-Cardiac surgery - Mortality	Shin S., 2021, South Korea[120] - Retrospective, Single Centres, 7629 pts	30 days Mortality due to Myocardial injury after non-cardiac surgery	1 - P	Preoperative medications, medical comorbidities, laboratory data	XGB, GBM, RF[*], SVM, KNN, LR - RF: AUC 0.927, F1-score 0.549	Internal validation, 5 fold cross validation	2	ASA, Possum score
Major elective non-Cardiac surgery - Mortality	Yun K., 2021, South Korea[118] - Retrospective, single centre, 1384 pts	Predicting unexpected postoperative mortality within 30 days of the surgery or within the same hospital stay as the surgery	1 - P	Demographics, laboratory results, disease-specific variables, type of surgery, operation name, operation time, blood loss, hemodynamic variables	NB, NN[*], DT, RF, Hellinger Distance DT, Hellinger Distance RF - NN: AUC 0.80, F1-score 0.83	Internal validation with ten-fold cross-validation	2	NA
Major elective non-Cardiac surgery - Procedural	Van de Sande D, 2021, Netherlands[131] - Retrospective, single center, 1174 pts	predict safe hospital discharge after the second postoperative day	1 - P	Demography, past medical history, diagnosis	RF - RF: AUROC 0.88, sensitivity 0.79, specificity 0.80	Internal validation, dataset divided in train, validate, and test	2	NA
Major elective non-Cardiac surgery - Procedural	Van de Sande D., 2022, Netherlands[43] - Retrospective, multiple centre, 1693 pts	Predict safe hospital discharge after the second postoperative day	2 - P	Demography, past medical history, diagnosis	RF (Van de Sande D., 2021) - RF: AUROC 0.83, sensitivity 0.78, specifitity 0.79	External validation, train (70%), validation (20%), and test data set (10%)	3	NA
Major elective non-Cardiac surgery - Procedural and Mortality	Chiew C. J., 2020, Singapore[114] - Retrospective, cohort, single centre, 90785 pts	Compare the performance of ML models against the traditional derived Combined Assessment of Risk Encountered in Surgery (CARES) and the American Society of Anaesthesiologists-Physical Status (ASA-PS) in predicting 30-day postsurgical mortality and need for intensive care unit > 24 hours	2 - P	Demographics, comorbidities, preoperative laboratory results, surgery type	RF, AdaBoost, GBM[*], SVM - Mortality - GBM: AUC of 0.96 - ICU Admission: GBM: AUC of 0.95, AUPRC:0.38	Internal validation with hold-out method, 30% of the database used for testing	2	American Society of Anaesthesiologists-Physical Status, Combined Assessment of Risk Encountered in Surgery
Major surgery non-Cardiac surgery - Morbidity	Dolendo I. M., 2022, USA[68] - Retrospective, cohort, single centre, 148 pts	To identify risk factors and develop machine-learning-based models to predict pts who are at higher risk for postoperative opioid use after mastectomy.	1 - P	Demographics, transgender, postmenopausal, ASA score, drug use, type of surgery	Multivariable LR, ridge regression, lasso, elastic net regression [*] - Elastic net regression: AUC of 0.80	Hosmer-Lemeshow to asses goodness-of-fit, internal validation by 10 fold cross-validation	2	Multivariable LR with variable selection used as a reference model
Major surgery non-Cardiac surgery - Morbidity	Zeng S., 2021, China[58] - Retrospective, single centre, 175 pts	Predict postoperative complication in liver resection patients	1 - P	Demographics, past medical history, bleeding, transfusion, duration of surgery, type of surgery, type of incision, tumour dimension and number	LR, Decision tree, SVM, RF - Decision tree C5.0: AUC 0.92, sensitivity 0.87, Specificity 0.94	Internal validation, 70/30 split	2	NA

Minor/moderate non-cardica surgery - Morbidity	Park K., 2022, South Korea[30] - Retrospective, single centre, 7833 pts	Prediction of aspiration pneumonia after gastric endoscopic submucosal dissection	1 - P	Demographics, ASA score, comorbidities, procedure time, sedation time, recovery time, location of the lesion, tumour size, haemodynamic during sedation, sedative drugs	multivariable LR, XGBoost[*], Elastic Net, RF, NN, SVM - XGBoost: AUC 0.74	Internal validation with hold-out method, 30% of the database used for testing	2	NA
Minor/moderate non-cardica surgery - Morbidity and Procedural	Gabriel R.A., 2022, USA[70] - Retrospective, single centre, 1333 pts	Develop machine learning algorithms incorporating pain and opioid features to predict the need for outpatient opioid refills following ambulatory surgery	1 - P	Demographics, past medical history, type of anaesthesia, type of surgery, pain score	LR, NN[*], SVM, RF, with and without SMOTE - NN with SMOTE: AUC 0.711, F1-score 0.347, sensitivity 0.693, specificity 0.979	Internal validation using k-fold cross validation	2	NA
Orthopedic Surgery - Morbidity	Castle J. P., 2022, USA[71] - Retrospective, single center, 581 pts	Identify prognostic factors for continued opioid use after arthroscopic meniscus surgery	1 - P	Demographics, comorbidities, procedure details, intra-operative Outerbridge Classification of arthritis severity	NB - NB: AUC 0.79, Brier score 0.12, sensitivity 0.95, PPV 0.83, detection rate 0.78, NPV 0.20, specificity 0.07	Internal validation, split 70/30	2	Univariate and multivariate Cox-regression model
Orthopedic Surgery - Morbidity	Gabriel R. A., 2022, USA[69] - Retrospective, cohort, single centre, 1042 pts	Predict the risk of persistent opioid use following lower extremity joint arthroplasty	1 - P	Demographics, preoperative opioid use, comorbidities, type of surgery, intraoperative medications, type of anaesthesia, postoperative medications	LR, BRF[*], BB[*], RF[*], MLP, SVM - BRF: AUC of 0.959, F1-score: 0.841, BB: AUC of 0.959, RF: AUC of 0.959	Internal validation with stratified 10-fold cross-validation	2	LR without SMOTE used as a reference
Orthopedic Surgery - Morbidity	Ko S., 2020, South Korea[40] - Retrospective, multicentre, 5757 pts	Prediction of Acute Kidney Injury after total knee arthroplasty	1 - P	Demographic, ASA score, comorbidities, preoperative medication, perioperative blood values, type of surgery, type of anaesthesia	GBM - GBM: AUC of 0.89, Sensitivity 0.65, Specificity 0.77	External Validation with third hospital dataset - Internal validation Stratified 10-fold cross-validation	3	NA
Orthopedic Surgery - Morbidity	Kunze K. N., 2022, USA[66] - Retrospective, multicentre, 616 pts	Prediciting all cause complications within two years of primary total hip arthroplasty (THA)	1 - P	Demographics, comorbidities, preoparitive modified Harris Hip Score, preoperative hipe flexion	Stochastic Gradient Boosting (SGB), RF, SVM, NN, LR - SGB: AUC 0.86, Brier Score 0.09	Internarnal validation with tenfold cross-validation	2	NA
Orthopedic Surgery - Morbidity	Zhao H., 2021, China[78] - Retrospective, single centre, 245 pts	Predicting delirium after elderly hip fracture surgeries.	1 - P	Demographics, comorbidities, preoperative values, surgery duration, type of anaesthesia	LR, RF, XGBoost, SVM, MLP - Accuracy for 4 models between 0.8a and 0.88, AUC reported only for LR: 0.78	Internal validation with 5-fold cross-validation	2	LR
Orthopedic Surgery - Mortality	Kitcharanant N., 2022, Thailand[116] - Retrospective, single centre, 492 pts	Predicting 1-year mortality after fragility hip fracture	1 - P	Demographics, type of fracture, type of treatment, comorbidities, time to surgery	GB, NN, RF[*], LR, SVM, NB, KNN - RF: AUC 0.95, sensitivity 0.68, specificity 0.99	Internal validation, splitting random ratio 70/30	2	NA
Orthopedic Surgery - Mortality	McLeod G., 2022, UK[117] - Retrospective, single centre, 329 pts	Creation of web-based dynamic nomogram to Predict 1 year mortalityl after hip fracture surgery	1 - P	Demographics, ASA score, preoperative blood values, socioeconomic status, length of surgery, procedure urgency, total time in theatre	Global Cox proportional hazards model using all available covariates, a final Cox proportional hazards model, GLM, LR - Cox proportional hazards model and logistic models showed good discrimination, with concordance index values of 0.732 and 0.781, respectively	Internal validation with Hold-out method, 30% of the Database used for testing	2	Nottingham Hip Fracture Score

Orthopedic Surgery - Procedural	Abbas A., 2022, Canada[130] - Retrospective, multiple centre from American College of Surgeons (ACS) National Surgical Quality Improvement Program database, 302.300 pts	Duration of surgery (DOS) and Length of Stay (LOS) after total knee arthroplasty	1 - P	Demographics, comorbidities, ASA score, transfusion, type of anesthesia, dyspnea	Linear, tree-based and multilayer perceptron (MLP) - MLP: mean square error for DOS 0.92 and LOS 0.72	The dataset was split into training, validation, and testing based on year. Hyperparamentes tuned on validation set.	2	Mean regressor
Orthopedic Surgery - Procedural	Ezuma C. O., 2022, USA[125] - Retrospective, multicentre data from NSQIP database, 1307 pts	To determine which ML is more precise in identifying the risk factors for unplanned overnight admission after knee surgery	1 - P	Demographics, ASA score, comorbidities, preoperative laboratory values, surgery characteristics	RF, XGBoost, AdaBoost, elastic net penalized LR, weighted ensemble of those 4 algorithms [*] - Ensemble of 4 algorithms: AUC of 0.722, results for other models not stated	Internal validation with bootstrapping with 1000 resamples.	2	LR
Orthopedic Surgery - Procedural	Gabriel R. A., 2019, USA[124] - Retrospective, single centre, 1018 pts	Prediction of length of Stay after total hip arthroplasty	1 - P	Demographics, comorbidities, perioperative opioid use, type of anaesthesia, surgical approach	LR[*], RF[*], KNN, SVM[*], Light GBM, XGBoost, MLP - best results for 3 different outcomes. Primary composite adverse outcomes - SVM: AUC of 0.716, ICU admission - LR: AUC of 0.791, prolonged length-of-stay - RF: AUC of 0.854	Internal validation with hold-out method, 30% of the database used for testing	2	NA
Orthopedic Surgery - Procedural	Li H., 2022, China[128] - Retrospective, single centre, 1826 pts	Prediction on length of stay post total knee arthroplasty	1 - P	Demographics, preoperative blood values, comorbidities, ASA score,, type of anaesthesia, operation duration	LR, XGBoost[*] - XGBoost: AUC 0.66 (14 features) and 0.74 (7 features)	Method of validation not stated	2	NA
Orthopedic Surgery - Procedural	Lu Y., 2020, USA[126] - Retrospective review of national surgical outcomes databases (National Surgical Quality Improvement Program - NSQIP), 4709 pts	Predict overnight hospital admission after anterior cruciate ligament reconstruction	1 - P	Demographics, ASA score, comorbidities, preoperative blood values, perioperative functional status, operation time	XGBoost, RF, AdaBoost, LDC and ensemble of 4 other models[*] - Ensemble: AUC 0.759	Internal validation with Monte Carlo cross-validation	2	NA
Orthopedic Surgery - Procedural	Song B. M., 2021, USA[127] - Retrospective, multicentre, 1276 pts	Identifying patient characteristics and risk factors for overnight admission, identifying pts requiring overnight admission	1 - P	Demographics, ASA score, comorbidities, medical history, preoperative laboratory values, surgery lenght, type of anaesthesia	GLM,RF, XGBoost, AdaBoost, Elastic-Net, weighted ensemble of the four algorithms [*] - Weighted ensemble of the four algorithms: 0.709	Internal validation with 10 fold cross validation with 3 repeats	2	Models compared with each other
Orthopedic Surgery - Procedural	Sridhar S., 2022, USA[129] - Retrospective, single centre, 158 pts	Predict Lenght of stay for total joint replacements	1 - P	Duke score, type of procedure, demographics, past medical history, socio-economics features	RF - Root mean square error: 0.7	Internal validation 80/20 split	2	NSQIP calculator
Spinal surgery - Morbidity and Procedural	Cabrera A., 2023, USA[62] - Retrospective, multi centre, 29949 pts	Predict and describe length of stay, readmission, reoperation, transfusion, and infection rates following elective posterior cervical decompression with instrumented fusion	1 - P	From ACS-NSQIP database, demographics, past medical history, surgery info, transfusion	RF - RF (transfusion): AUC 0.902, accuracy 0.76, sensitivity 0.88, specificity 0.75	Internal validation. Each model adopted a stratified tenfold cross-validation	2	NA

Spinal surgery - Procedural	Gabrial R.A., 2023, USA[132] - Retrospective, single centre, 3189 pts	Case duration spinal surgery	1 - P	Demographics, Past medical history, type of surgery, ASA score	LR (multivariable), RF, XGB regressor, KNN - XGB regressor: variance score of 0.778, an R 2 of 0.770, an RMSE of 92.95 minutes, and an MAE of 44.31 minutes.	Internal validation using k-fold cross validation	2	LR
Vascular surgery - Morbidity	Bai P., 2020, China[73] - Retrospective, single centre, 443 pts	Incidence of cerebral infarction and myocardial infarction	1 - P	Demographics, preoperative neurological symptoms, preoperative degree of vessel stenosis, intraoperative pressure, time of occlusion, shunt, LOS	XGBoost - Accuracy 0.94	Internal cross-validation. Patients negative for the outcome were used as control group	2	NA
Vascular surgery - Morbidity	Filiberto A. C., 2021, USA[84] - Retrospective, cohort, single centre, 1531 pts	Risk of postoperative acute kidney injury after major vascular surgery using MySurgeryRisk platform	1 - P	Demographics, comorbidities, ASA score, type of surgery	MySurgeryRisk algorithm (Feng Z) - MySurgeryRisk: AUC 0.77, accuracy 0.17, AUPRC 0.70	Internal validation with 1000 Bootstrap cohort	2	ASA score
Vascular surgery - Morbidity	Tan J., 2020, China[97] - Retrospective, single centre, 406 pts	Prediction of early phase postoperative hypertension (EPOH) requiring administration of intravenous vasodilators after carotid endarterectomy (CEA).	1 - P	Demographics, comorbidities, postoperative values	GBM - GBM: AUC 0.77, sensitivity 0.90, specificity 0.52	Internal validation with four-fold stratified cross	2	NA
