# Supplementary material for: Prediction of Complications and Prognostication in Perioperative Medicine: A Systematic Review and PROBAST Assessment of Machine Learning Tools
Source: Anesthesiology. Author manuscript; Available in PMC 2024 Jun 4. (PMC11146190; doi:10.1097/ALN.0000000000004764)
Supplement: Supplementary Information [file EMS191514-supplement-Supplementary_Information.pdf]

Downloaded from http://pubs.asahq.org/anesthesiology/article-pdf/doi/10.1097/ALN.0000000000004764/697055/aln.0000000000004764.pdf by Duke University–Durham, Editorial Office on 14 November 2023

Supplemental Digital Content

Supplemental Table 1. Summary of data extracted for each article included in the systematic review with a focus on features, outcomes and limitations stated, https://links.lww.com/ALN/D308.

Supplemental Table 2. Summary of Risk of Bias and Applicability Assessment for Different Domains According to the PROBAST, https://links.lww.com/ALN/D309.

Copyright © 2023 American Society of Anesthesiologists. All Rights Reserved. Unauthorized reproduction of this article is prohibited.

Downloaded from http://pubs.asahq.org/anesthesiology/article-pdf/doi/10.1097/ALN.0000000000004764/697055/aln.0000000000004764.pdf by Duke University--Durham, Editorial Office on 14 November 2023

Appendix

The following research query was conducted: ((((“artificial intelligence”[All Fields]) OR (“machine learning” [All Fields])) AND (“perioperative” [All Fields])) OR (“surgery” [All Fields])) OR (“anaesthesia” [All Fields])) OR (“preoperative” [All Fields])))). In addition to the systematic review, manual searches were performed using the main research query and one or more of the following terms: AND pneumonia OR chest infection, AND myocardial infarction OR heart failure, AND sepsis, AND acute kidney injury, AND delirium OR stroke, AND infection, AND intubation, AND length of stay, AND bleeding, AND ileus, AND pain, AND complication, AND wound infection, AND skin and soft tissue infection, AND readmission, AND urinary tract infection, AND hypotension, AND transfusion, AND surgical duration, AND post operative venue, AND neural networks, AND extreme gradient boosting, AND random forest, AND support vector machine, AND NPL, AND generative AI.

Copyright © 2023 American Society of Anesthesiologists. All Rights Reserved. Unauthorized reproduction of this article is prohibited.
