# Supplementary material for: Prediction of Complications and Prognostication in Perioperative Medicine: A Systematic Review and PROBAST Assessment of Machine Learning Tools
Source: Anesthesiology. 2023 Nov 9;140(1):85–101. doi: 10.1097/ALN.0000000000004764 (PMC11146190; doi:10.1097/ALN.0000000000004764)
Supplement: Supplementary file 1 [file aln-140-85-s002.pdf]

Supplementary Table 2 : Summary of Risk of Bias and Applicability Assessment for Different Domains According to the PROBAST Tool.

**Author, year, country**	ROB - Partecipant	ROB - Predictors	ROB - Outcome	ROB - Analysis	Applicability - Partecipants	Applicability - Predictors	Applicability - Outcome	ROB	Applicability
Abbas A., 2022, Canada[130]	+	-	+	+	+	+	+	-	+
Adhikari L., 2019, USA[87]	-	?	+	+	+	-	+	-	-
Awadalla S. S., 2022, USA[67]	+	-	-	-	+	-	-	-	-
Bai P., 2020, China[73]	?	-	?	?	+	-	+	?	-
Bertsimas D., 2018, USA[63]	-	?	+	?	+	+	+	?	+
Bihorac A., 2019, USA[98]	-	+	+	+	+	-	+	-	-
Bishara A., 2021, USA[81]	-	?	+	+	+	+	+	-	+
Bishara A., 2022, USA[76]	-	+	+	+	+	+	+	-	+
Bolourani S., 2020, USA[83]	-	?	-	-	+	+	+	-	+
Bonde A., 2021, USA[36]	-	-	-	-	+	+	+	-	+
Brennan M., 2019, USA[33]	+	+	+	-	+	+	+	-	+
Cabrera A., 2023, USA[62]	+	+	?	?	+	+	+	?	+
Cartailler J., 2019, France[100]	+	+	-	-	+	-	+	-	-
Castela Forte J., 2021, Netherland[110]	-	-	+	+	+	-	+	-	-

Castela Forte J., 2022, Netherland[111]	-	+	+	+	+	-	+	+	-
Castle J. P., 2022, USA[71]	+	-	-	+	+	+	+	-	+
Chae D., 2022, South Korea[61]	-	?	+	+	+	+	+	-	+
Chelazzi C., 2021, Italy[54]	-	+	+	-	+	+	+	-	+
Chen C, 2023, China[91]	+	+	+	-	+	+	+	+	+
Chen C., 2021, China[37]	-	+	+	-	+	+	+	-	+
Chen P.F., 2022, Taiwan [121]	-	+	+	?	+	-	+	-	-
Chiew C. J., 2020, Singapore[114]	-	+	+	-	+	+	+	+	+
COVIDSurg Collaborative, 2021 ,international panel[123]	+	+	+	-	-	+	-	+	-
Dolendo I. M., 2022, USA[68]	-	+	?	?	+	+	-	?	-
Du Y., 2022, China[94]	-	-	-	-	-	+	+	-	-
El Hechi M.W, 2021, USA[39]	+	?	+	?	+	+	+	?	+
Ezuma C. O., 2022, USA[125]	+	+	?	?	+	-	-	?	-
Fernandes M. P. B., 2021, USA[48]	-	?	?	-	+	-	+	?	-
Filiberto A. C., 2021, USA[84]	-	+	+	?	+	+	+	-	+
Fritz B. A., 2019, USA[113]	-	+	+	+	+	+	+	-	+

Gabrial R.A., 2023, USA[132]	-	?	+	-	-	-	+	?	-
Gabriel R. A., 2019, USA[124]	-	+	-	+	+	+	+	-	+
Gabriel R. A., 2022, USA[69]	-	+	+	+	+	+	+	-	+
Gabriel R.A., 2022, USA[70]	-	-	-	?	+	-	-	?	-
Gao J., 2021, USA[122]	-	-	+	+	+	+	+	-	+
Gao Y., 2022, China[64]	-	-	-	-	+	+	+	-	+
Hatib F., 2018, USA[27]	+	+	?	?	+	-	+	+	-
Hill B. L., 2019, USA[115]	-	+	+	?	+	+	+	?	+
Hofer I.S., 2020, USA[53]	-	-	?	+	+	+	+	-	+
Hu X. Y., 2021, China[77]	+	+	-	+	+	-	+	+	-
Jacquet-Lagreze M., 2019, France[101]	+	+	+	?	+	+	+	?	+
Jeong Y. S., 2021, South Korea[55]	-	+	+	?	+	+	+	?	+
Jiang H., 2021, China[56]	-	+	+	?	+	+	+	-	+
Jones R. N., 2021, USA[79]	+	+	?	?	+	+	+	?	+
Jungquist C. R., 2019, USA[109]	-	+	+	?	+	+	+	?	+
Kamaleswaran R., 2021, USA[92]	-	+	+	?	+	-	+	-	-

Kang A. R., 2020, South Korea[102]	-	?	+	?	+	+	+	?	+
Karri R., 2021, Australia[72]	+	+	+	?	+	?	+	+	?
Kendale S., 2018, USA[106]	-	+	+	-	+	+	+	-	+
Kitcharanant N., 2022, Thailand[116]	-	?	+	?	+	+	+	?	+
Ko S., 2020, South Korea[40]	-	+	+	+	+	+	+	+	+
Koretsky M., 2022, USA[52]	+	-	-	+	+	+	+	-	+
Kristinsson ÆÖ, 2022, Denmark[26]	+	+	?	-	+	+	+	?	+
Kunze K.N., 2022, USA[66]	+	-	-	-	+	+	+	-	+
Lee C. K., 2021, USA[119]	-	+	+	-	+	+	+	-	+
Lee H. C., 2018, South Korea[85]	-	?	+	?	+	?	+	?	?
Lee H. C., 2018, South Korea[86]	-	+	+	?	+	+	+	?	+
Lee S., 2021, South Korea[105]	-	+	+	+	+	-	+	-	-
Lee SW, 2022, South Korea[112]	-	+	-	+	+	+	+	-	+
Li H., 2022, China[128]	-	+	+	?	+	+	+	?	+
Li P., 2023, China[93]	+	-	+	?	+	+	+	?	+
Li T., 2022, China[88]	-	-	+	?	+	?	+	?	?

Li X. F., 2021, China[50]	?	?	?	?	+	?	+	?	?
Lu Y., 2020, USA[126]	?	+	?	+	+	+	+	?	+
Lundberg S. M., 2018, USA[108]	?	+	+	-	+	+	+	-	+
MacKay E., 2021, USA[41]	+	+	+	?	+	+	+	-	+
Maheshwari K., 2020, USA[104]	+	+	+	-	+	-	+	-	-
Mascarella M. A., 2022, Canada[60]	+	+	?	?	+	+	+	?	+
McLeod G., 2022, UK[117]	?	+	+	?	+	+	+	?	+
Misic V., 2020, USA[42]	-	+	+	+	+	+	+	+	+
Nudel J., 2021, USA[59]	+	+	-	+	+	+	+	-	+
Oh A., 2023, South Korea[74]	-	?	+	?	+	+	+	?	+
Park K., 2022, South Korea[30]	-	+	+	?	+	+	+	-	+
Peng X., 2022, China[75]	+	+	+	?	+	+	+	-	+
Petrosyan Y., 2022, Canada[89]	-	+	+	?	+	+	+	-	+
Racine A. M., 2020, USA[80]	+	+	+	-	+	+	+	-	+
Ren W., 2022, China[95]	?	?	?	?	+	?	+	?	?
Schenk J., 2021, Netherlands[103]	+	?	+	+	+	?	+	-	?

Shi S., 2021, China[90]	-	+	+	?	+	+	+	-	+
Shin S., 2021, South Korea[120]	-	+	?	?	+	-	+	?	-
Sobrie O., 2016, Belgium[49]	-	+	+	-	+	+	+	-	+
Solomon S. C., 2020, USA[107]	-	+	?	?	+	+	+	?	+
Song B. M., 2021, USA[127]	?	+	?	-	+	+	+	?	+
Song Y., 2023, China[82]	-	+	?	-	+	+	+	?	+
Sridhar S., 2022, USA[129]	-	+	-	?	+	+	+	-	+
Suhre W., 2020, USA[31]	+	?	?	-	+	+	+	?	+
Tan J., 2020, China[97]	?	+	?	?	+	?	+	?	?
Tavolara T. E., 2021, USA[34]	+	?	+	-	+	?	+	-	?
Tourani R., 2019, USA[32]	+	+	+	-	+	+	+	+	+
Tschoellitsch T., 2022, Austria[96]	-	+	+	?	+	+	+	-	+
Van de Sande D, 2021, Netherlands[131]	?	-	+	-	+	+	-	?	-
Van de Sande D., 2022, Netherlands[43]	?	-	-	-	+	+	-	-	-
Walczak S., 2020, USA[44]	+	+	?	+	+	+	+	?	+
Wijnberge M., 2020, Netherlands[35]	+	+	?	?	+	?	+	?	?

Xie T., 2021, China[99]	+	?	?	-	+	-	+	?	-
Xue B., 2021, USA[57]	-	+	+	+	+	+	+	-	+
Xue Q., 2021, China[65]	-	+	+	-	+	+	+	-	+
Yun K., 2021, South Korea[118]	+	+	?	-	+	+	+	?	+
Zeng S., 2021, China[58]	-	+	-	-	+	+	+	-	+
Zhang L., 2021, China[51]	?	+	+	-	+	?	+	-	?
Zhang Y., 2021, China[28]	-	+	?	-	+	+	+	?	+
Zhao H., 2021, China[78]	+	+	+	+	+	+	+	+	+
